# Typical and disrupted brain circuitry for conscious awareness in full-term and preterm infants

**DOI:** 10.1093/braincomms/fcac071

**Published:** 2022-03-24

**Authors:** Huiqing Hu, Rhodri Cusack, Lorina Naci

**Affiliations:** 1 Trinity College Institute of Neuroscience, School of Psychology, Trinity College Dublin, Dublin, Ireland; 2 Global Brain Health Institute, Trinity College Dublin, Dublin, Ireland

**Keywords:** neonate, premature birth, brain development, high-order networks, conscious awareness

## Abstract

One of the great frontiers of consciousness science is understanding how early consciousness arises in the development of the human infant. The reciprocal relationship between the default mode network and fronto-parietal networks—the dorsal attention and executive control network—is thought to facilitate integration of information across the brain and its availability for a wide set of conscious mental operations. It remains unknown whether the brain mechanism of conscious awareness is instantiated in infants from birth. To address this gap, we investigated the development of the default mode and fronto-parietal networks and of their reciprocal relationship in neonates. To understand the effect of early neonate age on these networks, we also assessed neonates born prematurely or before term-equivalent age. We used the Developing Human Connectome Project, a unique Open Science dataset which provides a large sample of neonatal functional MRI data with high temporal and spatial resolution. Resting state functional MRI data for full-term neonates (*n* = 282, age 41.2 weeks ± 12 days) and preterm neonates scanned at term-equivalent age (*n* = 73, 40.9 weeks ± 14.5 days), or before term-equivalent age (*n* = 73, 34.6 weeks ± 13.4 days), were obtained from the Developing Human Connectome Project, and for a reference adult group (*n* = 176, 22–36 years), from the Human Connectome Project. For the first time, we show that the reciprocal relationship between the default mode and dorsal attention network was present at full-term birth or term-equivalent age. Although different from the adult networks, the default mode, dorsal attention and executive control networks were present as distinct networks at full-term birth or term-equivalent age, but premature birth was associated with network disruption. By contrast, neonates before term-equivalent age showed dramatic underdevelopment of high-order networks. Only the dorsal attention network was present as a distinct network and the reciprocal network relationship was not yet formed. Our results suggest that, at full-term birth or by term-equivalent age, infants possess key features of the neural circuitry that enables integration of information across diverse sensory and high-order functional modules, giving rise to conscious awareness. Conversely, they suggest that this brain infrastructure is not present before infants reach term-equivalent age. These findings improve understanding of the ontogeny of high-order network dynamics that support conscious awareness and of their disruption by premature birth.

## Teaser

Hu *et al.* report that the default mode and fronto-parietal networks are instantiated in infants by full-term age, but not before. The reciprocal relationship between them is also present by this age, regardless of prematurity. Findings improve understanding of the ontogeny of network dynamics that support conscious awareness.

## Graphical Abstract

**Graphical Abstract:**
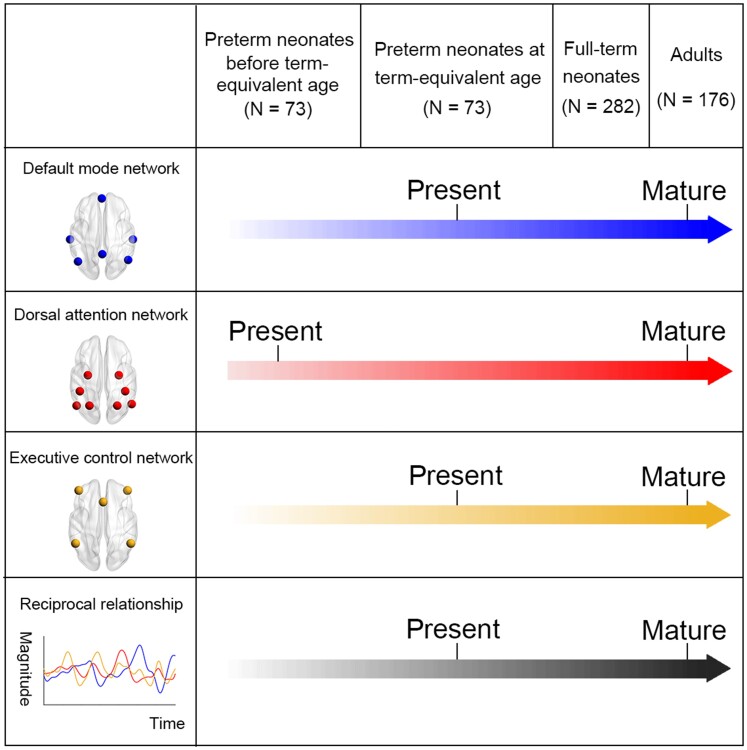

## Introduction

It remains unknown whether conscious awareness is present in newborn infants and whether its development is affected by premature birth. In healthy human adults, consciousness is clinically defined and measured by examining two distinct components: arousal and awareness.^1^ ‘Arousal’ is measured by assessing spontaneous eye opening, sleep-wake cycles and other systemic fluctuations in the ability to engage with the environment. ‘Awareness’ is assessed by examining the ability to wilfully respond to commands behaviourally and/or through language, as well as the ability to report on mental states pertaining to oneself or the environment.^2–4^ There is no question that newborn infants, or neonates, have arousal, e.g. they cry and have sleep-wake cycles. However, the extent to which neonates have awareness, or can consciously process information about themselves and their environment remains unknown, and is central to this study.

Although studies on neonate awareness are scarce, a few suggest ‘minimal’ awareness from birth. Newborns perform some forms of stimulus discrimination early after birth, including distinguishing their body (12 to 103 h after birth),^5^ and their own cry from those of other newborns (within 1 day after birth),^6,7^ their mother’s voice from a stranger’s (within 12 h—3 days after birth)^8–10^ and discriminating facial expressions of happiness from disgust (2 days after birth).^11^ Although prima facia these studies suggest ‘minimal’ awareness, these behaviours could be due to certain stimuli being primed in the early days of life or even in the womb or due to the physical properties of the stimuli themselves. For example, the mother’s voice is very familiar to the neonate, so any preferential responses could be due to familiarity rather than understanding the meaning and significance of the mother figure. Similarly, the response to expressions of disgust could be a pre-conscious reaction to aversive stimuli.^12^ Critically, the lack of language and the very limited motor function preclude self-report or behavioural responses and, thus, prevent the assessment of infant awareness from the first days of life. To circumvent these limitations, in the present study, we followed a different strategy.

We asked a foundational question to understand the *capacity* for conscious experience that of whether or not the brain mechanisms of conscious awareness are instantiated in neonates. One of the most influential theories of consciousness, the Global Neuronal Workspace Theory, postulates that consciousness requires integration of information from discrete but interconnected modules across the brain.^13–15^ Adult functional neuroimaging studies have identified the fronto-parietal and default mode network (DMN) networks as two such distinct cortical systems that support consciousness and play complementary roles in information integration. Myriad neuro-psychological and neuroscientific studies show that fronto-parietal regions—comprising the dorsal attention (DAN) and executive control (ECN) networks—are critical for stimulus-driven high-order cognition,^16–20^ and recent work suggests they facilitate awareness of external stimuli.^21–23^ By contrast, the DMN has been primarily implicated in self-referential and self-awareness processing,^22–31^ and more recently in context processing.^32–34^ Importantly, the fronto-parietal network and DMN share a reciprocal relationship, where they are not simultaneously active, i.e. are anticorrelated, or exhibit a low correlation of functional time-courses relative to other brain network pairings. This relationship is abolished when consciousness is extinguished, irrespective of condition, e.g. whether during deep anaesthesia under various pharmacological manipulations or after severe brain injury,^21,35,36^ suggesting that it tracks the presence/absence of conscious awareness, as dissociable from arousal (such as, in the case of wakeful brain injured patients in the vegetative state).^35^

Whether the DMN, DAN and ECN and their reciprocal relationship are developed by birth and whether they are affected by premature birth and neonate age remain poorly understood and are the focus of this study. To assess the literature, we conducted a literature search with key words, ‘functional network’ or ‘functional connectivity’, ‘infant’ or ‘newborn’ or ‘neonatal’ and ‘fMRI’ that resulted in 20 neonate studies summarized in Supplementary Table 1. Whilst the three primary sensory and motor networks were consistently reported in neonates,^37–42^ findings were inconsistent on the presence of high-order networks, including the DMN, DAN and ECN. Some resting state functional magnetic resonance imaging (rs-fMRI) studies found no evidence for the presence of these networks until the end of the first year.^38,42–44^ Fransson *et al*.^42^ found that the DMN in preterm neonates was fragmented into an anterior and posterior part. Similarly, Gao *et al*.^43^ reported that although the two main hubs of DMN (i.e. the ventral/dorsal medial prefrontal cortex and posterior cingulate/retrosplenial cortex) were consistently observed in 2-week-olds, 1-year-old and 2-year-olds, other aspects of the DMN (i.e. the inferior parietal lobule, lateral temporal cortex and hippocampus regions) were not found in 2-week-olds. A similar pattern was also reported for the DAN and ECN.^39,41^ By contrast, other rs-fMRI studies support the idea that these networks have already emerged in neonates.^40,45–47^ For instance, Doria *et al*.^40^ found that both primary and high-order networks were present in full-term and preterm neonates scanned at term-equivalent age (TEA: 37–42 weeks of postmenstrual age). He *et al*.^47^ detected a fronto-parietal network, comprising the frontal gyrus and inferior parietal cortex, and a second one, comprising the anterior cingulate cortex, medial prefrontal cortex, superior/middle frontal gyrus, in preterm neonates. Linke *et al*.^45^ found that both the ECN and DMN were present even in neonates with perinatal brain injuries, both full-term and preterm neonates scanned at TEA. Eyre *et al*.^48^, the largest study of newborn brain network topography to date, used the Developing Human Connectome Project (dHCP) dataset and reported an adult-like topography in six high-order networks, including a fronto-parietal network similar to the DAN distribution, but did not find evidence for the DMN. This study did not investigate the reciprocal relationship between these networks. The extant evidence for the emergence of the DMN, DAN and ECN at birth is summarized in Supplementary Table 2.

Several factors may contribute to these divergent results. Due to methodological and technical challenges, the incipient field of infant neuroimaging has, to date, not adhered to unified testing protocols. The aforementioned studies employ vastly different sample sizes (e.g. ranging from *n* = 11 to 143), different MR field strengths (e.g. 1.5T versus 3T) yielding different spatial and temporal resolutions,^49,50^ different motion artefact control methods and lack age-specific structural brain templates for neonates. The multitude of different methodologies across previous studies renders it impossible to conclude, in light of inconsistent results, whether the DMN, DAN and ECN are already present at birth or not. Moreover, to the best of our knowledge, only one study to date has investigated whether the reciprocal relationship between these three high-order networks is developed in early infancy.^51^ Gao *et al*.^51^ reported that the anticorrelated interaction between the DMN and DAN was absent at birth but became apparent at one year of age. However, this study had a relatively small number of full-term neonates (*n* = 51) and no preterm neonates, which may have reduced the power to detect effects of interest. To address the aforementioned limitations of previous studies, we used the open-source dHCP dataset, which conferred several advantages, including a large sample size (*n* = 282), 3T magnetic resonance imaging (MRI), multiband echo-planar imaging (EPI) that significantly improves temporal resolution and signal-to-noise,^52^ registration to more accurate week-to-week neonate structural templates, and significant improvements in motion correction and signal-to-noise ratio relative to previous studies (see Methods for further details).

We included neonates delivered and scanned at full-term (*n* = 282) and a reference adult group (*n* = 176). To investigate the effect of neonate age on these networks and their relationship, we also included preterm neonates. The effect of chronological age at the time of assessment was deconfounded from the effect of premature birth,^53^ by the inclusion of two groups: the first (*n* = 73) born prior to but scanned at TEA, and the second (*n* = 73) born and scanned before TEA. A subset of paired scans collected from the same infants (*n* = 37) before and at TEA was also included. We reasoned that any differences between neonates born and scanned at full-term and preterm neonates scanned at TEA would reflect effects of premature birth, while controlling for neonate age. Conversely, any differences between preterm neonates scanned at TEA and those scanned before TEA would reflect the effects of neonate age. First, we investigated the development of the DMN, DAN and ECN and of their reciprocal relationship in each neonate group. We then tested the hypothesis that premature birth and early neonate age are associated with altered network architecture.

## Materials and methods

### Participants

#### Neonates

**Figure 1:**
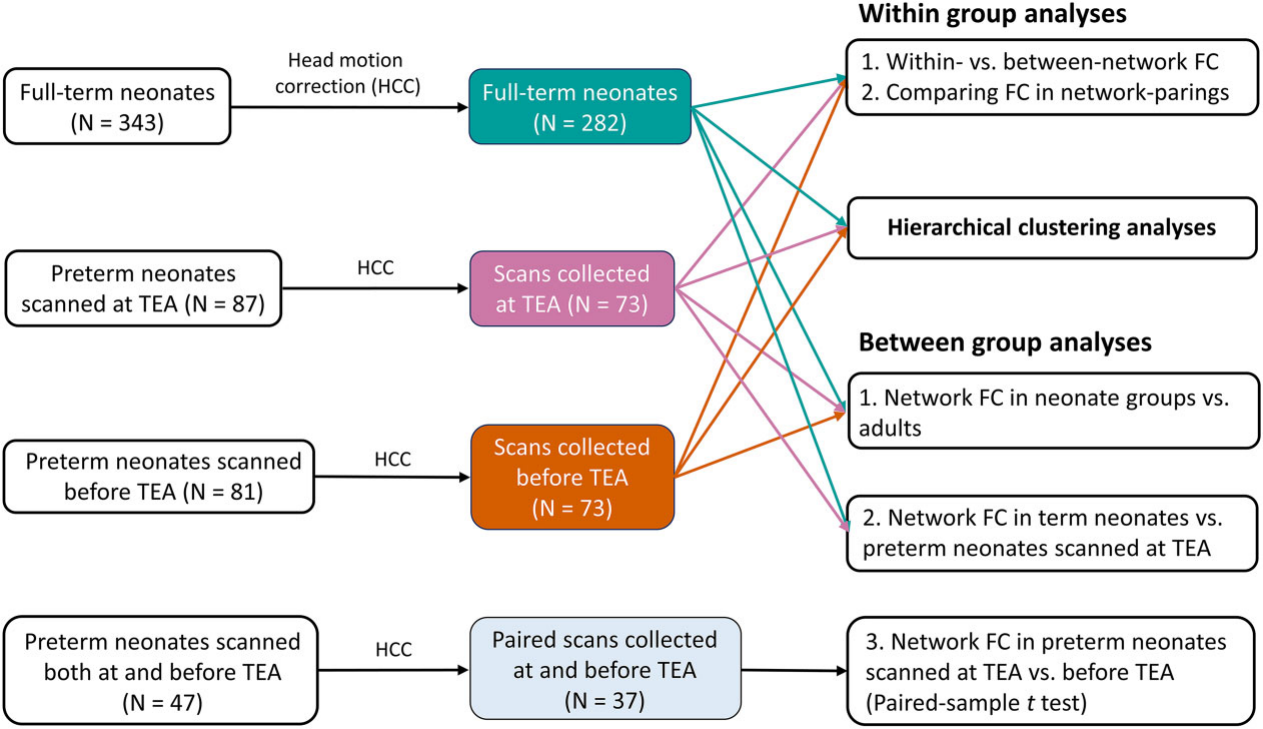
**The number of scans included in data analyses**. From top to bottom, the middle column indicates the number of scans that passed head motion criteria in each neonate group. TEA, term-equivalent age; vs., versus; FC, functional connectivity.

The neonate data were from the second (2019) dHCP public data release (http://www.developingconnectome.org/second-data-release/). All neonates were scanned at the Evelina Newborn Imaging Centre, Evelina London Children’s Hospital. Ethical approval was obtained from the UK’s National Research Ethics Committee, and parental informed consent was obtained prior to imaging. *Full-term neonates*. We used 282/343 scans in the full-term neonates [gestational age (GA) at birth = 40.0 weeks ± 8.6 days; postmenstrual age (PMA) at scan = 41.2 weeks ± 12.0 days; 160 males] after quality control procedures. *Preterm neonates*. We used 73 scans in both the preterm neonates scanned at TEA (GA at birth = 32.0 weeks ± 25.6 days; PMA at scan = 40.9 weeks ± 14.5 days; 41 males) and preterm neonates scanned before TEA (GA at birth = 32.5 weeks ± 13.4 days; PMA at scan = 34.6 weeks ± 13.4 days; 50 males). Of the 47 preterm neonates scanned both at and before TEA, 10 were discarded because of excessive movement of either one of the two scans, resulting in 37 paired scans (GA at birth = 31 weeks ± 6.8 days; PMA at first scan = 34 weeks ± 1.3 days; PMA at second scan: 40 weeks ± 6.9 days; 24 males). Further details are in Fig. 1 and SI file and Supplementary Table 3.

#### Adults

As a reference adult group, we used a subset (*n* = 176; 22–36 years; 77 males) of high-quality data from the final release of the Washington University-Minnesota Consortium of Human Connectome Project (HCP) selected by Ito *et al*.^54^ (https://github.com/ito-takuya/corrQuench). For details of study procedures see Van Essen *et al*.^55^

### Data acquisition and pre-processing

#### dHCP

Data were acquired on a 3T Philips Achieva with a dedicated neonatal imaging system including a neonatal 32-channel phased-array head coil. Fifteen minutes of high temporal and spatial resolution rs-fMRI data were acquired using a multislice gradient-echo EPI sequence with multiband excitation (TE = 38 ms; TR = 392 ms; MB factor = 9×; 2.15 mm isotropic, 2300 volumes). In addition, single-band EPI reference (sbref) scans were also acquired with bandwidth-matched readout, along with additional spin-echo EPI acquisitions with 4×AP and 4×PA phase-encoding directions. To correct susceptibility distortion in rs-fMRI data, field maps were also obtained from an interleaved (dual TE) spoiled gradient-echo sequence (TR = 10 ms; TE1 = 4.6 ms; TE2 = 6.9 ms; flip angle = 10°; 3 mm isotropic in-plane resolution, 6 mm slice thickness). High-resolution T1- and T2-weighted anatomical imaging were also acquired in the same scan session, with a spatial resolution of 0.8 mm isotropic. For T1w image: TR = 4795 ms and the field of view (FOV) = 145 × 122 × 100 mm. For T2w image: TR = 12 000 ms, TE = 156 ms and the FOV = 145 × 122 × 100 mm.

The dHCP rs-fMRI data were pre-processed by dHCP group using the project’s in-house pipeline optimized for neonatal imaging. See SI and Fitzgibbon *et al*.^56^ for full details. In order to reduce signal artefacts related to head motion, the cardiorespiratory fluctuations and multiband acquisition, the 24 extended rigid-body motion parameters together with single-subject independent component analysis (ICA) noise components were regressed out. To further reduce the effect of motion on functional connectivity (FC) measures, motion-outlier volumes were identified, and a scrubbing procedure was applied to retain a continuous sub-sample of the data (∼70%) with the lowest motion for each participant. The subjects who still had a high level of motion after scrubbing procedure were excluded from further analyses. We discarded the first 5 volumes to allow for adaptation to the environment and equilibrium of the MR signal at first. Then, motion outliers were identified from the remaining 2295 volumes. Volumes with the root mean square intensity difference between successive volumes higher than 1.5 interquartile range above the 75th centile, after motion and distortion correction, were considered motion outliers. Then, a continuous sub-sample of 1600 volumes with the minimum number of motion outliers was retained for each subject. Subjects with more than 160 motion-outlier volumes (10% of the cropped dataset) in the continuous subset were labelled ‘high level of motion’ and excluded entirely. Thus, 8 preterm neonates scanned before TEA, 14 preterm neonates scanned at TEA and 61 full-term neonates were excluded. In addition, we performed a temporal low-pass filter (0.08 Hz low-pass cutoff) on the pre-processed dHCP rs-fMRI to conduct FC analyses, as previous studies found that oscillations were primarily detected within grey matter in 0.01–0.08 Hz.^57,58^Supplementary Fig. 1A provides a schematic of the processing steps for dHCP fMRI data.

#### HCP

Data were acquired on a customized 3T Siemens ‘Connectome Skyra’ with a 32-channel head coil. Resting state images were collected using gradient-echo EPI sequence: TR = 720 ms; TE = 33.1 ms; flip angle = 52°; FOV = 208 × 180 mm (RO × PE), slice thickness = 2 mm, 72 slices, 2.0 mm isotropic voxels, 1200 volumes per run. rs-fMRI data were pre-processed by HCP group. See SI and Van Essen *et al*.^55^ for full details.

### Data analyses

#### Network definition

We used a theory and meta-analyses driven node-based approach to network mapping. Nineteen regions of interest (ROIs) (8-mm radius spheres) for the three networks (Supplementary Table 4), DMN, DAN and ECN, were created based on well-established landmark ROIs defined in Raichle.^59^ This method also helps to relate findings to our previous findings based on the same parcellation template.^35,60^ See SI file, Supplementary Figs. 2 and 3 for details of alignment to neonate week-to-week structural templates and full details.

#### FC

FC between ROIs was assessed by calculating the Pearson correlation of pre-processed time-courses, and z scored by using the Fisher-*z* transformation. At the individual level, within-network FC was obtained by averaging the FC between ROIs belonging to the same network and between-network FC by averaging the FC between ROIs of each network to the others. For all between-group comparisons, the ROI-level FC within each subject was normalized to facilitate a focus on the FC-patterns between groups rather than potentially differing FC strength between the groups.^48,61^ Analysis of variances (ANOVAs) and *t*-tests were used to explore within and between-group differences. Bonferroni correction for multiple comparisons was applied to all statistical results.

#### Comparison of neonates and adults

General linear models (GLMs) were used to test for group differences in FC within DMN, DAN or ECN while controlling for head motion, as we found neonates had significantly higher head motion than adults (SI, Supplementary Figs. 4 and 5). Hierarchical clustering analysis and non-metric multidimensional scaling were used to capture network structure, and visualize the similarity of ROI responses in neonates and adults.^62,63^ See SI for further details.

#### Comparison of neonate groups

To investigate the effect of premature birth, while controlling for age at scan, on network development, we compared the FC within each network and between each pair of networks, between preterm neonates scanned at TEA and full-term neonates. Head motion was not included as a covariate, since we did not observe any significant difference between the two groups (Supplementary Fig. 5). To investigate the effect of neonate age, while controlling for prematurity, the same preterm neonates scanned before and at TEA (*n* = 37) were used.

### Data availability

Raw neonate data can be obtained from the Developing Human Connectome Project website (http://www.developingconnectome.org/second-data-release/) and raw adult data can be obtained through the Washington University-Minnesota Consortium of Human Connectome Project website (http://www.humanconnectomeproject.org). Derived data not published will be available upon reasonable request.

## Results

### The development of high-order networks in neonates

**Figure 2:**
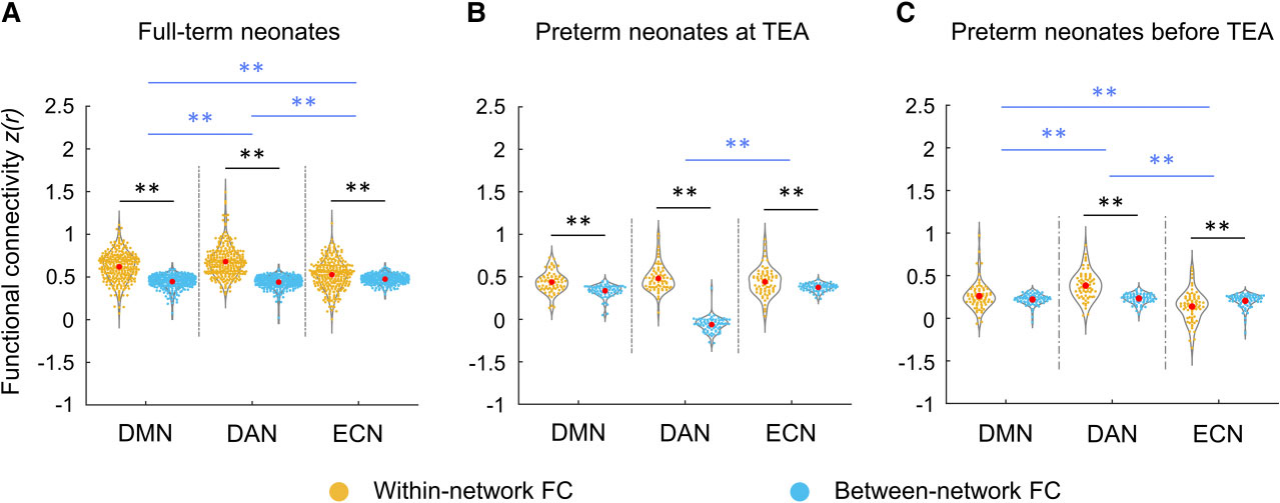
**Within-network and between-network FC across DMN, DAN and ECN in the neonate groups.** (**A**) full-term neonates; (**B**) preterm neonates scanned at TEA; (**C**) preterm neonates scanned before TEA. The black lines/asterisks indicate significant difference between FC measures for each network (Paired *t*-tests), and the blue lines/asterisks indicate significant difference in FC measures of different networks (Paired *t*-tests). The FC values were Fisher-*z* transformed and inter-subject variability was removed for display purposes. ** = *P* < 0.005.

For full-term neonates, a 2 × 3 repeated measure ANOVA [type of FC (within-network, between-network) ×  network (DMN, DAN, ECN)] showed a significant main effect of type of FC [*F* (1, 281) = 766.14, *P* < 0.001], which was driven by higher overall connectivity for the within- relative to between-network connectivity (*t* (281) = 27.63, *P* < 0.001) (Fig. 2A). We also found a main effect of network [*F* (2, 562) = 16.29, *P* < 0.001], which was driven by lower overall connectivity for the ECN relative to the DMN [*t* (281) = − 3.06, *P* < 0.005] and DAN [*t* (281) = −5.84, *P* < 0.001]. A significant interaction effect of type of FC by network [*F* (1.96, 550.18) = 81.44, *P* < 0.001] was driven by smaller difference between within- and between-network connectivity in the DMN relative to the DAN [*t* (281) = −4.41, *P* < 0.001), and in the ECN relative to the DMN [*t* (281) = −8.88, *P* < 0.001], and the DAN [*t* (281) = −11.86, *P* < 0.001]. Paired *t*-tests showed significantly higher within- to between-network FC for each network [DMN: *t* (281) = 17.32, *P* < 0.001; DAN: *t* (281) = 21.05, *P* < 0.001; ECN: *t* (281) =  5.51, *P* < 0.001] (Fig. 2A). This suggested that the coherence of nodes within each network was stronger than with the other networks’ nodes, in other words, that each of the three networks was differentiated as a cohesive unit, distinct from the other networks.

For preterm neonates scanned at TEA, a similar 2 × 3 repeated measure ANOVA showed a significant main effect of FC type [*F* (1, 72) = 87.04, *P* < 0.001], which was driven by higher overall connectivity within- relative to between-network connectivity [*t* (72) = 9.35, *P* < 0.001] (Fig. 2B). A significant interaction effect of type of FC by network [*F* (1.81, 130.23) = 6.03, *P* < 0.005] was driven by a smaller difference between within- and between-network connectivity in ECN relative to the DAN [*t* (72) = −2.96, *P* < 0.005]. Paired *t*-tests showed significantly higher within- relative to between-network FC for each network [DMN: *t* (72) = 6.20, *P* < 0.001; DAN: *t* (72) =  8.05, *P* < 0.001; ECN: *t* (72) = 3.46, *P* < 0.001] (Fig. 2B), suggesting that the DMN, DAN and ECN were distinct from one another in preterm neonates scanned at TEA.

For preterm neonates scanned before TEA, a similar 2 × 3 repeated measure ANOVA showed a significant main effect of type of FC [*F* (1, 72) = 16.80, *P* < 0.001], which was driven by higher overall connectivity for the within- relative to between-network connectivity [*t* (72) = 4.12, *P* < 0.001] (Fig. 2C). A main effect of network (*F* [1.83,131.64) = 22.15, *P* < 0.001] was driven by lower overall connectivity for the DMN [*t* (72) = −3.98, *P* < 0.001] and ECN [*t* (72) = −6.45, *P* < 0.001] relative to the DAN (Fig. 2C). A significant interaction effect of type of FC by network [*F* (2, 144) = 33.78, *P* < 0.001] was driven by smaller difference between within- and between-network connectivity in DMN relative to that in DAN [*t* (72) = −3.93, *P* < 0.001], and in ECN relative to that in DMN [*t* (72) = −4.32, *P* < 0.001] and DAN [*t* (72) = −8.21, *P* < 0.001]. Paired *t*-tests showed significantly higher within- relative to between-network FC for DAN [*t* (72) = 7.73, *P* < 0.001] but significantly lower within-network FC compared to between-network FC for ECN [*t* (36) = −3.86, *P* < 0.001] (Fig. 2C), suggesting that only the DAN was distinct from the other two networks in preterm neonates scanned before TEA.

In summary, these results suggested that the DMN, DAN and ECN were present in full-term and preterm neonates scanned at TEA, but only the DAN was formed as a distinct network in the preterm neonates scanned before TEA. Furthermore, the DAN was the most cohesive network in all three neonate groups.

### The development of the reciprocal relationship between the DMN and fronto-parietal networks in neonates

**Figure 3:**
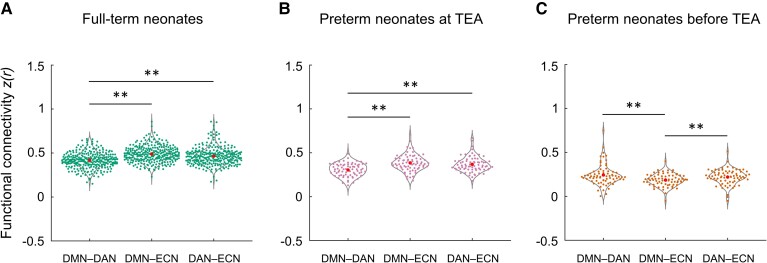
**Between-network functional connectivity in neonate groups.** (**A**) full-term neonates; (**B**) preterm neonates scanned at TEA; (**C**) preterm neonates scanned before TEA. One-way ANOVAs with repeated measures and Paired *t*-tests were used here. The FC values were Fisher-*z* transformed and inter-subject variability was removed for display purposes. DMN−DAN, FC between the DMN and DAN; DMN−ECN, FC between the DMN and ECN; DAN−ECN, FC between the DAN and ECN; **P < 0.005.

In full-term neonates, a one-way ANOVA with repeated measures for between-network FC (DMN−DAN, DMN−ECN, DAN−ECN) showed a significant main effect [*F* (1.98, 555.60) = 27.11, *P* < 0.001], which was driven by significantly lower FC in the DMN−DAN relative to DMN−ECN [*t* (281) = −7.79, *P* < 0.001] and DAN−ECN [*t* (281) = −4.64, *P* < 0.001] pairings (Fig. 3A). Similarly, to the adult data (see SI Results, Supplementary Fig. 7), the lower DMN−DAN FC, relative to the other pairings suggested that the reciprocal relationship between the DMN and DAN was present in full-term neonates. In preterm neonates scanned at TEA, a similar main effect (*F* [1.87, 134.45) = 11.93, *P* < 0.001] was driven by significantly lower FC in the DMN–DAN compared to DMN–ECN [*t* (72) = −4.78, *P* < 0.001] and DAN–ECN [*t* (72) = −4.20, *P* < 0.001] pairings (Fig. 3B) and suggested that the reciprocal relationship between the two networks was present in preterm neonates scanned at TEA. In preterm neonates scanned before TEA, a significant main effect [*F* (2, 144) = 4.86, *P* = 0.009] was driven by lower FC in DMN−ECN relative to DMN−DAN [*t* (72) = −2.88, *P* = 0.005] and DAN−ECN [*t* (72) = −2.94, *P* < 0.005] pairings (Fig. 3C). This is consistent with the aforementioned results suggesting that the DMN and ECN are not yet developed as distinct networks in this group (Fig. 2C). Furthermore, these results suggested that, by contrast to the full-term and preterm neonates scanned at TEA, the relationships between the three networks in preterm neonates scanned before TEA do not yet resemble the adult pattern (see SI, Supplementary Fig. 7). In summary, these results suggested that the reciprocal relationship between the DMN and DAN has started to develop in full-term neonates and preterm neonates scanned at TEA, but not in preterm neonates scanned before TEA.

### Comparison of neonate and adult networks

**Figure 4:**
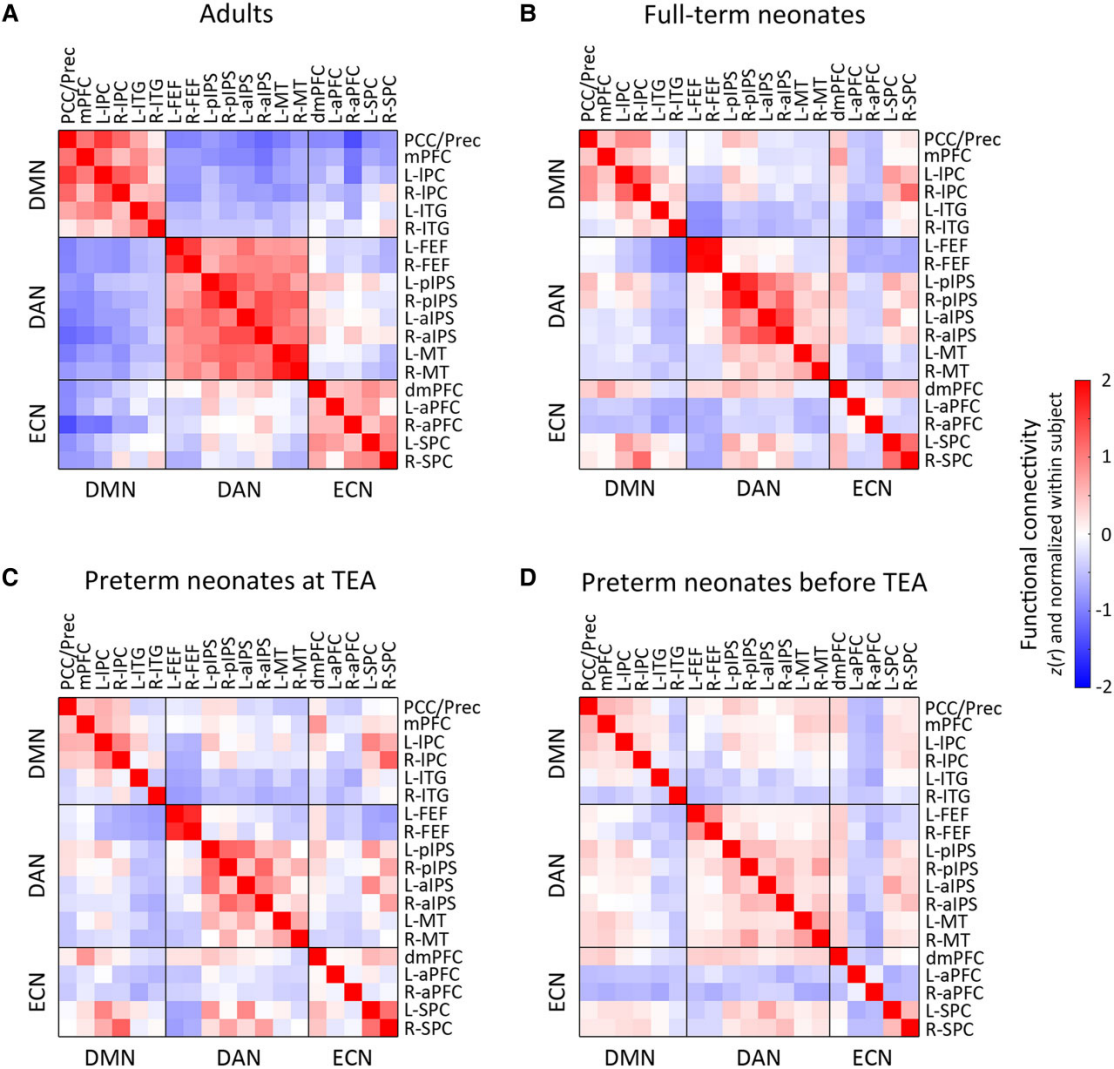
**FC in adults and neonate groups.** (**A**) adults, (**B**) full-term neonates, (**C**) preterm neonates scanned at TEA and (**D**) preterm neonates scanned before TEA. The FC value presents here was Fisher-*z* transformed and normalized within each subject before being averaged within each group. R, right; L, left; PCC/Prec, posterior cingulate cortex/precuneus; mPFC, medial prefrontal cortex; lPC, lateral parietal cortex; ITG, inferior temporal gyrus; FEF, frontal eye field; pIPS, posterior intraparietal sulcus; aIPS, anterior intraparietal sulcus; MT, middle temporal area; dmPFC, dorsal medial prefrontal cortex; aPFC, anterior prefrontal cortex; SPC, superior parietal cortex.

**Figure 5:**
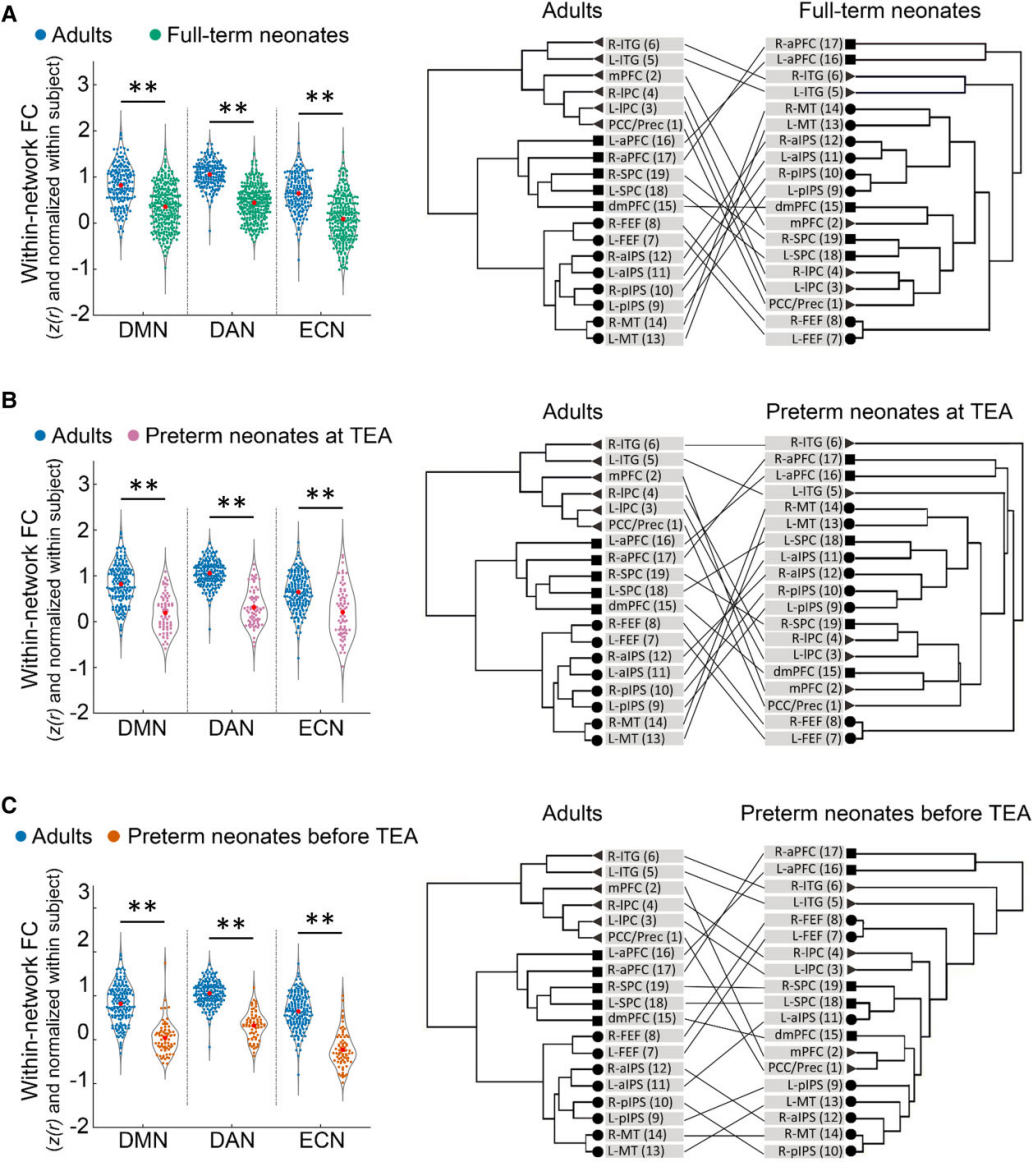
**The development of the DMN, DAN and ECN in neonates relative to adults.** (**A**) full-term neonates, (**B**) preterm neonates scanned at TEA and (**C**) preterm neonates scanned before TEA relative to the adults. The left panels of (**A**), (**B**) and (**C**) depict the comparison of within-network FC between each neonate group and the adults. GLMs were used to test for group differences while controlling for head motion. The FC values were Fisher-*z* transformed and normalized within each subject before being averaged within each group. The right panels of (**A**), (**B**) and (**C**) depict the network structure of each neonate group relative to adults. Triangles/circles/squares represent the nodes of the DMN/DAN/ECN. R, right; L, left; PCC/Prec, posterior cingulate cortex/precuneus; mPFC, medial prefrontal cortex; lPC, lateral parietal cortex; ITG, inferior temporal gyrus; FEF, frontal eye field; pIPS, posterior intraparietal sulcus; aIPS, anterior intraparietal sulcus; MT, middle temporal area; dmPFC, dorsal medial prefrontal cortex; aPFC, anterior prefrontal cortex; SPC, superior parietal cortex. **P < 0.005.

**Figure 6:**
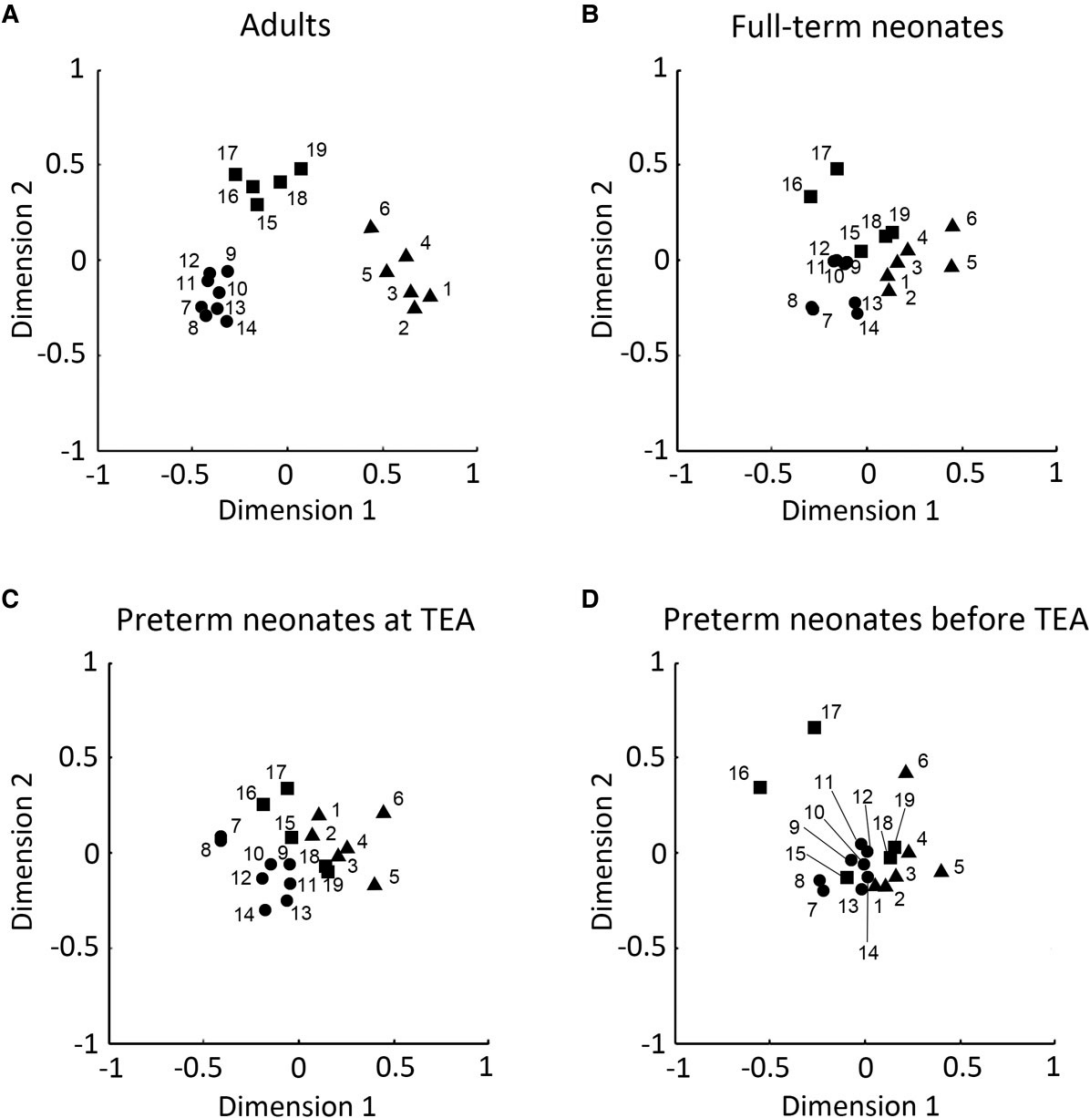
**Multidimensional scaling (MDS) plots of regions in adults and neonates**. (**A**) adults, (**B**) full-term neonates, (**C**) preterm neonates scanned at TEA and (**D**) preterm neonates scanned before TEA. The 2-D plots were created using non-metric MDS based on node’s similarity. Here triangles/circles/squares indicate nodes of the default mode/dorsal attention/executive control network. Abbreviations: 1, posterior cingulate cortex/precuneus; 2, medial prefrontal cortex; 3, left lateral parietal cortex; 4, right lateral parietal cortex; 5, left inferior temporal gyrus; 6, right inferior temporal gyrus; 7, left frontal eye field; 8, right frontal eye field; 9, left posterior intraparietal sulcus; 10, right posterior intraparietal sulcus; 11, left anterior intraparietal sulcus; 12, right anterior intraparietal sulcus; 13, left middle temporal area; 14, right middle temporal area; 15, dorsal medial prefrontal cortex; 16, left anterior prefrontal cortex; 17, right anterior prefrontal cortex; 18, left superior parietal cortex; 19, right superior parietal cortex; TEA, term-equivalent age.

**Figure 7:**
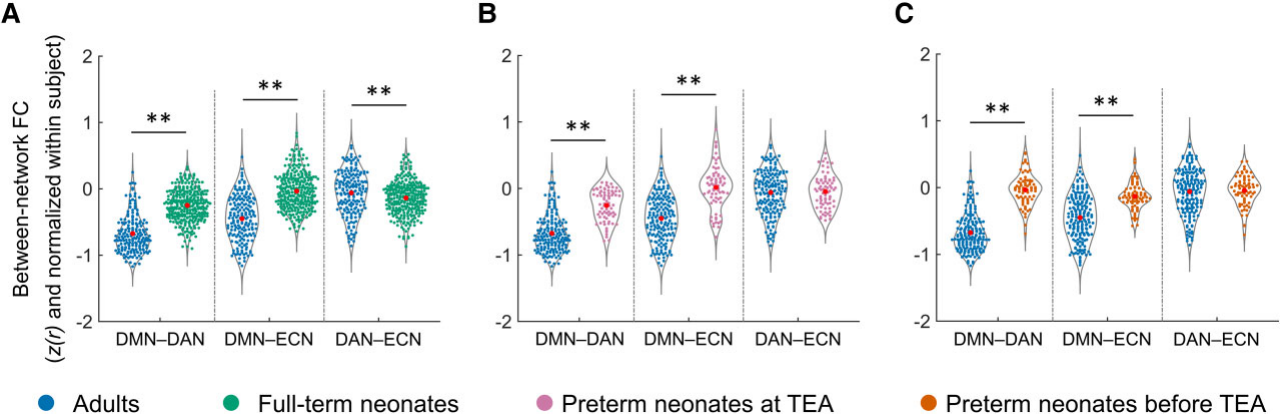
**The development of the between-network FC in neonates relative to adults**. (**A**) full-term neonates, (**B**) preterm neonates scanned at TEA and (**C**) preterm neonates scanned before TEA relative to the adults. GLMs were used to test for group differences while controlling for head motion. The FC values were Fisher-*z* transformed and normalized within each subject before averaging within each group. Abbreviations: FC, functional connectivity; DMN−DAN, FC between DMN and DAN; DMN−ECN, FC between DMN and ECN; DAN−ECN, FC between DAN and ECN; *P* < 0.005.

Visual inspection of the connectivity matrices (Fig. 4) suggested that each neonate group had less cohesive networks (lower within relative to between-network connectivity) than the adult group. To investigate specifically how the three networks in neonates differed from those of adults, we compared the within- (Figs. 5 and 6) and between-network (Fig. 7) connectivity in the neonate and adult groups.

A GLM comparing adults and full-term neonates, including head motion as a covariate (SI, Supplementary Fig. 5), showed a significant main effects of group for all of the three networks [DMN: *F* (1, 455) = 75.62, *P* < 0.001; DAN: *F* (1, 455) = 333.33, *P* < 0.001; ECN: *F* (1, 455) = 135.88, *P* < 0.001; Fig. 5A], which was driven by significantly higher within-network FC in the adults relative to full-term neonates. Hierarchical clustering analyses showed that, the adults’ network nodes grouped neatly into the a-priory postulated three distinct clusters,^59^ each comprising all the ROIs belonging to that network (Supplementary Table 4). By contrast, in the full-term neonates, the ROIs clustered into groups that were inter-mixed between the three networks (Fig. 5A). Similarly, for the preterm neonates scanned at TEA, we found significant main effects of group for all of the three networks [DMN: *F* (1, 246) = 90.16, *P* < 0.001; DAN: *F* (1, 246) = 252.32, *P* < 0.001; ECN: *F* (1, 246) = 42.40, *P* < 0.001; Fig. 5B], again driven by significantly higher within-network FC in the adult group. Unlike the adult group, preterm ROIs clustered into groups inter-mixed between the three networks (Fig. 5B). Consistent with the other two groups’ results, for the preterm neonates scanned before TEA, we found significant main effects of group for all ofthe three networks [DMN: *F* (1, 246) = 145.15, *P* < 0.001; DAN: *F* (1, 246) =  276.15, *P* < 0.001; ECN: *F* (1, 246) = 218.91, *P* < 0.001; Fig. 5C], which were driven bysignificantly higher within-network FC in adults, and ROI clusters that did not adhere to network identity (Fig. 5C).

Multidimensional scaling analyses further confirmed these results, by showing that the adult ROIs formed three distinct clusters conforming to network identify (Fig. 6A), distanced from one another in representational space. By contrast, each network’s ROIs in the neonate groups formed less distinct clusters, i.e. clusters were more closely grouped together, and their separability as distinct clusters was reduced with neonate age. The preterm neonates scanned before TEA showed the most intermingling of ROIs across the three networks in this 2D manifold (Fig. 6D).

In summary, these results suggested that although the three networks were present in full-term and preterm neonates scanned at TEA, and the DAN in preterm neonates scanned before TEA, their coherence was lower and structure less well-organized than the canonical adult networks. This is consistent with previous studies showing that brain networks continue to develop from birth onwards.^40,64–67^ As expected, the reciprocal relationship between the DMN and fronto-parietal networks was also weaker in neonates relative to adults (Fig. 7) (see SI Results for full details).

### The effect of premature birth on network development and their relationship

**Figure 8:**
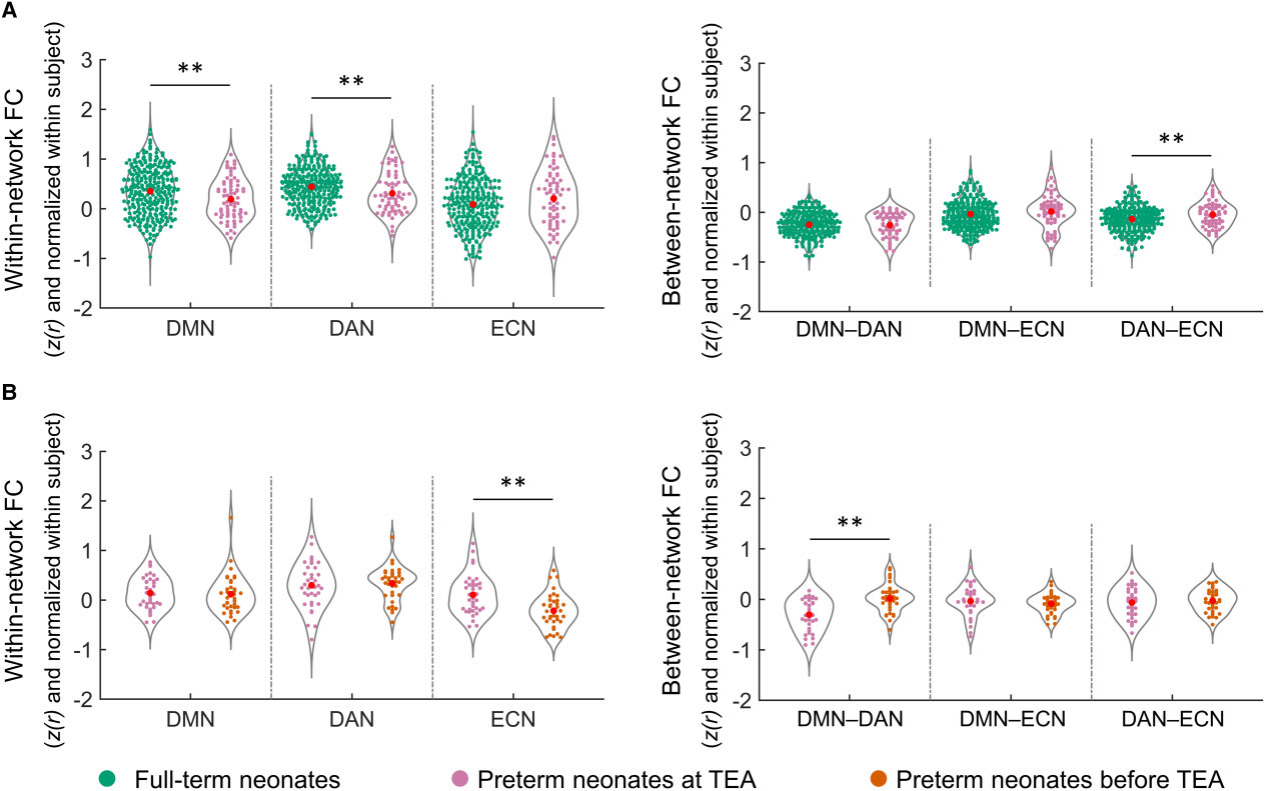
**The effect of premature birth and early neonate age on the development of network FC.** (**A**) The effect of premature birth on within-network FC (the left panel) and between-network FC (the right panel). The FC values represented in (**A**) were Fisher-*z* transformed and normalized within each subject before averaging within each group. Independent-sample *t*-tests were applied to detect the effect of premature birth here. (**B**) The effect of early neonate age on within-network FC (the left panel) and between-network FC (the right panel). The FC values represented in (B) were Fisher-z transformed, normalized within each subject, and inter-subject variability was removed for display purposes. Paired *t*-tests were applied to detect the effect of early neonate age here. Abbreviations: DMN, default mode network; DAN, dorsal attention network; ECN, executive control network; DMN−DAN, FC between DMN and DAN; DMN−ECN, FC between DMN and ECN; DAN−ECN, FC between DAN and ECN; **P < 0.005.

Comparison of network FC in full-term relative to preterm neonates scanned at TEA showed significantly lower FC within the DMN [*t* (353) = −2.64, *P* = 0.009] and the DAN [*t* (353) = −2.63, *P* = 0.009] in the preterm group (Fig. 8A). In addition, we observed higher FC between the DAN and ECN [*t* (353) = 2.83, *P* = 0.005] in preterm neonates (Fig. 8A). These results suggested that, by TEA, premature birth is associated with lower DMN and DAN network coherence, and lower differentiation of the DAN and ECN from one another, relative to full-term birth.

### The effect of neonate age on network development and their relationship

We found significantly lower FC within the ECN [*t* (36) = −4.28, *P* < 0.001] and higher DMN–DAN FC [*t* (36) = 4.39, *P* < 0.001], suggesting lower ECN network coherence and lower functional differentiation between the DMN and DAN, for preterm neonates scanned before TEA relative to this same group of infants scanned once they reached TEA(Fig. 8B). Thus, these results suggested that neonate age, and particularly, development up to TEA, is a significant factor for the maturation of the ECN and of the development of the reciprocal relationship between the DMN and the DAN.

## Discussion

In this study, we asked whether infants have the capacity for conscious experiences. The lack of language and willful motoric output presents major barriers to consciousness science in neonates. To sidestep these limitations, we examined whether or not the brain circuitry of conscious awareness is developed at birth. In particular, we focused on the impact of premature birth and early neonate age on the development of the default mode and fronto-parietal networks and of their reciprocal relationship. The critical novel contribution of this study is showing that the DAN, ECN and DMN are already present in neonates by full-term or TEA, and furthermore, that the reciprocal relationship between the DMN and the DAN, is already instantiated by this age. By contrast, this relationship is not present in preterm neonates before TEA.

### Effect of premature birth

We found that the three networks were present in preterm neonates at TEA. Furthermore, for the first time, we show that the reciprocal relationship between the DAN and DMN was instantiated in preterm neonates at TEA. Previous rs-fMRI neonate studies that have investigated the effect of premature birth have mainly focused on disrupted within-network coherence or topological organization,^48,61,68,69^ and knowledge of the impact of premature birth on the relationship between high-order brain networks remains scarce. It is, therefore, striking to see that this key functional relationship develops in healthy-born premature neonates by TEA, according to a pre-programmed developmental trajectory despite of prematurity.

However, premature birth had a negative impact on network development. Preterm neonates at TEA had significantly lower within-network connectivity in the DMN and DAN, suggesting that these networks were less developed relative to full-term neonates despite the matched age. Furthermore, the DAN–ECN connectivity was higher in preterm neonates at TEA, likely due to weaker within-network connectivity in the DAN. Our results are consistent with Smyser *et al*.^61^ and Eyre *et al*.^48^ findings of lower within-network connectivity in preterm relative full-term neonates. Bouyssi-Kobar *et al*.^70^ showed decreased density of connections in parietal-temporal and frontal areas in preterm neonates scanned at TEA relative to full-term neonates, using graph theoretical modelling. Previous structural MRI studies also found widespread deficiencies in grey and white matter,^71,72^ including in the DMN regions, in preterms scanned at TEA relative to full-term neonates.^71^ Consistent with a growing literature, our results shed light on disrupted brain mechanisms that may underlie the significant risks for neurodevelopmental and psychiatric problems in later life,^53,73–75^ that are associated with premature birth.

### Impact of neonate age on network development

In contrast to preterm neonates assessed at TEA, neonates who were assessed before TEA showed dramatic underdevelopment of networks and their relationships. Only the DAN, but not the ECN and DMN, was present as a discrete network. Strengthening of within-network connectivity in fronto-parietal regions has been reported in previous studies that assessed preterm neonates longitudinally.^46^ These findings suggest that, relative to other high-order networks, e.g. the DMN, the ECN aspect of the fronto-parietal networks is the least developed in preterms and most liable to undergo developmental change in the early weeks post birth. Furthermore, the reciprocal relationship between the fronto-parietal and DMN networks also strengthened in the weeks up to TEA. It was not formed before TEA, but appeared once preterms reached TEA. These results present a novel finding for premature neonates. They resonate with fetal studies showing that coactivation from the posterior cingulate cortex (node of the DMN) to areas of dorsal attention/executive networks became more negatively coupled with increasing fetal age.^76,77^

### Comparison of neonate and adult networks

On the one hand, it is important to note that although the DAN, ECN and DMN were already present at full-term and TEA, they were significantly different from the adult networks. The neonate networks had significantly lower within-network connectivity and were atypical in their nodal structure, suggesting less within-network cohesiveness compared to the adults. Similarly, the reciprocal relationship between the DMN and DAN was less developed in neonates relative to adults. These findings are consistent with previous studies showing that the functional organization of the brain rapidly develops from birth onwards.^43,78,79^ For example, Gao *et al*.^43^ reported that the DMN becomes adult-like by 2 years of age. A developmental shift towards the adult pattern of high FC variability in high-order networks has been reported for the DAN and fronto-parietal networks, which can be predicted by developmental cortical expansion.^80^ Studies have also reported significant differences in segregation and integration, indicators of functional network maturation, in the DMN and ECN between childhood and adulthood.^78,79,81^

Of the three high-order networks, the ECN was the least developed in premature neonates, whereas the DAN was the most well-formed across all neonate groups. These results suggest different ontogenesis trajectories for the two fronto-parietal networks, likely explained by their differing functions. The DAN serves to orient and modulate attention to the saliency of incoming sensory inputs,^82^ a capacity that probably emerges early in fetal development, as foetuses start to perceive sounds inside the womb. By contrast, behavioural response planning and monitoring, subserved by the ECN,^83^ is a mental faculty that relies heavily on the increasingly complex interactions with their environment that neonates engage in as they mature from the first weeks and months after birth.

On the other hand, our results show a strikingly similar patterning of the neonatal brain to the adult brain, with three key high-order brain networks and the reciprocal relationship between them being present by TEA. Our results in full-term neonates resonate with previous studies that observed high-order networks in this group.^40,45–47,84^ Our results are also consistent with some brain structural studies, suggesting that fundamental structural features of the adult brain are already present in neonates from birth. For example, the white matter microstructure, a putative neural basis for information processing speed and intelligence in adults, is present in very early life.^85^ Furthermore, distinct hierarchical tiers of network nodes resembling those of the adult brain have been found in neonates,^86^ and graph theoretical measures have shown an efficient rich club organization of the neonatal brain structure similar to that observed in adults.^87^

### Methodological considerations

Although some previous studies have observed high-order networks in neonates,^40,45–47,84^ others have not.^38,39,42^ This inconsistency is likely due to methodological differences in the type of data acquired and the analyses method. By contrast to some previous studies,^38,39,42^ here we were enabled by the high quality dHCP dataset to employ a uniquely large sample of high temporal and spatial resolution infant rs-fMRI data, and accurate week-by-week structural templates of the developing infant brain, which ensured higher sensitivity to detect brain networks in early infancy. Second, we used a theoretically motivated ROI analysis rather than a data-driven ICA for network definition. While ICA is a convenient data-driven tool to extract networks, it requires a critical free parameter that determines how many will be extracted, and hence the degree to which brain networks are likely to be detected as whole versus broken into parts. For example, a recent paper by Eyre *et al*.^48^, that used the dHCP dataset, did not find a single network corresponding to the whole DMN, but it is possible that this result would have changed with a different parameter choice. Given these methodological strengths and clear results, we believe this study helps to resolve previous inconsistent findings on the development of high-order brain networks in neonates. We note that the preterm group is not well representative of extreme prematurity (<28 weeks)/very low birth weight (<1500 g), because the mean GA of the preterm group was 32 weeks. Extreme prematurity could be associated with different functional architectures at TEA, as well as during the preterm period, compared with infants with higher GAs, and will be investigated in future studies.

### Implications for understanding conscious awareness in neonates

What are the implications of our findings for understanding the conscious experiences of neonates? Highly relevant to understanding neonate awareness is Damasio’s^88^ distinction between a ‘core awareness’, or a basic integrated experience of the current moment, and an ‘extended awareness’, made possible by the accumulation of autobiographical memories that allow creation of an internal world and projection beyond the present.^88^ This is echoed in the distinction between a ‘minimal’ self—an immediate^89^ form that comprised awareness of body boundaries, position, facial features, visceral states, etc,^90^—and a ‘longitudinal’ self, which requires the presence of episodic autobiographical memory and semantic self-knowledge, and is extended across time.^91^ While some studies suggest a ‘minimal’ awareness, the presence of ‘longitudinal’ awareness from birth has, to date, has remained unknown.

For the first time, here we show that by full-term birth or TEA, neonates possess key features of the brain infrastructure that enables the integration of information across diverse sensory and higher order functional modules, which gives rise to conscious awareness. This is consistent with a recent study that shows processing of cross-modal stimuli and learning of multimodal contingencies in neonates.^92^ We also show that this system is yet to undergo substantial change before it resembles that of the adults. Therefore, while these findings suggest that the capacity for conscious experiences is present at birth, coupled with previous evidence, they suggest that such experiences may be limited. The frontal cortex, a nexus of the fronto-parietal and DMN networks, undergoes dramatic maturation and reorganization by the end of the first year of life, resulting in big improvements in several cognitive abilities, and in sophistication of related mental content at that age.^93^ Consistent with neuroanatomical data, Kouider *et al*.^94^ found an electrophysiological signature of perceptual consciousness which was present albeit weak and delayed in 5-month-olds, became stronger and faster in 12−15-month-old infants. Kovacs *et al*.^95^ found that infants’ eye movements demonstrated a capacity to monitor other people’s beliefs at 7 months, and to make predictions about visual scenes at 12 months. Critically, at birth, neonates are largely bereft of the wealth of prior experiences that inform episodic memory and semantic knowledge, which allow for the construction of longitudinal awareness,^88,91^ and the creation of an internal world that projects beyond the present and is extended across time. Nevertheless, even from the first days of life, neonates encode, store, and retrieve information about events in their world.^96–98^ They start to integrate sensory, kinesthetic and proprioceptive stimulus response contingencies, in order to understand the actions of others and generate models for producing similar actions. Our results suggest that from birth neonates possess the capacity to integrate sensory and incipient cognitive experiences into coherent conscious experiences about their core self and the developing relationship to their environment.

## Supplementary Material

fcac071_Supplementary_Data

## Abbreviations

DAN =: dorsal attention network
dHCP =: the Developing Human Connectome Project
DMN =: default mode network
DVARS =: the root mean square intensity difference between successive volumes
ECN =: executive control network
FC =: functional connectivity
GA =: gestational age
HCP =: the Human Connectome Project
ICA =: independent component analysis
PMA =: postmenstrual age
ROIs =: regions of interest
rs-fMRI =: resting-state functional magnetic resonance imaging
TEA =: term-equivalent age.
