# Supplementary material for: Typical and disrupted brain circuitry for conscious awareness in full-term and preterm infants
Source: Brain Commun. 2022 Mar 24;4(2):fcac071. doi: 10.1093/braincomms/fcac071 (PMC9006044; doi:10.1093/braincomms/fcac071)
Supplement: fcac071_Supplementary_Data [file fcac071_supplementary_data.pdf]

Supplementary Information

Typical and disrupted brain circuitry for conscious awareness in full-term and preterm infants

Huiqing Hu[1], Rhodri Cusack[1], Lorina Naci[1,2*]

Author affiliations:

[1] Trinity College Institute of Neuroscience, School of Psychology, Trinity College Dublin, Dublin, Ireland.

[2]Global Brain Health Institute, Trinity College Dublin, Dublin, Ireland

[*]**Correspondence to:**

Lorina Naci

School of Psychology

Trinity College Institute of Neuroscience

Global Brain Health Institute

Trinity College Dublin

Dublin, Ireland

Telephone: +353 (0)87 688 5642

Email: nacil@tcd.ie

Supplementary Methods

Participants

Full-term neonates: Data from 342 full-term neonates were available. Only one subject was scanned twice, at 37 weeks postmenstrual age (PMA) and after 37 weeks PMA, but we only used the later one for the full-term group. 61 scans were discarded because of excessive movement (see Data Analyses section). Thus, the full-term neonate group had 282 participants (gestational age (GA) at birth = 40.0 weeks ± 8.6 days; PMA at scan = 41.2 weeks ± 12.0 days; 160 males).

Preterm neonates. 121 preterm neonates had fMRI data in the second dHCP public data release. 74 of them were scanned once: 40 participants scanned at TEA and 34 participants scanned before TEA. 47 of the 121 preterm neonates were scanned twice, at and before TEA. Thus, for preterm neonates, we have 87 obtained at TEA and 81 before TEA. 14/87 scans collected at TEA and 8/81 scans collected before TEA were discarded because of excessive movement (see Data Analyses section).

Adults. All participants were right-handed, native English speakers, and had no history of neurological disorders. All participants were tested at Washington University in accordance with the protocol approved by the Washington University institutional review board. Informed consent was obtained for each participant prior to the experiment. Specific details and procedures of subject recruitment can be found in Van Essen et al.[1]. The subset used in the current study passed stringent quality control measures relative to the larger HCP.[2] Detailed information about exclusion criteria can be found in Ito et al.[2] and the full list of the 176 participants used in this study is available here: https://github.com/ito-takuya/corrQuench.

Data Acquisition

dHCP data acquisition. Full details of dHCP data acquisition can be found at Fitzgibbon et al.[3]. Prior to scanning, neonates were fed, swaddled, and comfortably positioned in a vacuum jacket to promote natural sleep.

HCP. Full details of HCP data acquisition can be found at Van Essen et al.[1]. Rs-fMRI data were collected in four runs of 14.4 minutes each, two runs in one session and two in another session. The two sessions were conducted in two days separately and we only used the data collected in the first session. Within each session, oblique axial acquisitions alternated between phase encoding in a right-to-left (RL) direction in one run and phase encoding in a left-to-right (LR) direction in the other run.

Data pre-processing

dHCP. The dHCP rs-fMRI data were pre-processed by dHCP group using the project's in-house pipeline optimized for neonatal imaging and specifically developed for this dataset, detailed in Fitzgibbon et al.[3]. This pipeline includes: 1) Motion and distortion correction: corrects for intra-volume movement artefacts and for artefacts associated with susceptibility-induced off-resonance field changes (susceptibility-by-movement artefacts), and estimates motion nuisance regressors; 2) Registration: aligns all functional images with the native T2 space and the neonatal template space, which refers to the week-specific 40-week template from the dHCP volumetric atlas[4]; 3) Temporal high-pass filter: 150s high-pass cut-off; 4) Denoising: Estimates artefact nuisance regressors and regresses all nuisance regressors from the functional data obtained from the first step.

HCP. The rs-fMRI data of HCP were pre-processed by HCP group using the following pipeline: 1) Distortion correction: correction of gradient-nonlinearity-induced distortion and

phase-encoding-direction-induced distortion; 2) Motion correction: realigns the timeseries to correct for subject motion by using a 6 DOF FLIRT (Oxford Centre for Functional MRI of the Brain Fs Linear Registration Tool) registration of each frame to the single-band reference image; 3) Aligns the original EPI data (rs-fMRI data) to Montreal Neurological Institute (MNI) template space: EPI to T1w from FLIRT BBR, fine tuning of EPI to T1w with BBR-register, nonlinear T1w to MNI template; 4) Intensity normalization to mean of 10000 and bias field removal; 5) Temporal high-pass filter: 150s high-pass cut-off; 6) Denoising: removes artefactual or "bad" components using ICA-FIX to automatically. Detailed pre-processing procedure can be found in Glasser et al.[5]. Additionally, we performed a temporal low-pass filter (0.08 Hz low-pass cut-off) on the denoised rs-fMRI data and removed the first five volumes. Supplementary Fig. 1 provides a schematic of the processing steps for HCP fMRI data. As the selection of the subset of HCP had controlled head motion (i.e., exclusion of participants that had any fMRI run in which more than 50% of TRs had greater than 0.25mm framewise displacement), and adults generally have smaller maximal head motion than neonates,[6] we did not apply the same scrubbing method used in the dHCP dataset to adult data. To assess the effect of this, we compared the mean framewise displacement (FD) value in adults and neonates before/after the scrubbing procedure.[7,8] The FD value indexes the movement of the head from one volume to the next and is defined as the sum of the absolute values of the differential realignment estimates (the six realignment parameters). It has been widely used to index head movement and exclude subjects of high motion.[9-11] Independent-samples *t*-tests showed that adults had significantly lower head motion compared to neonates before (t (491.45) = -12.49, $p < 0.001$) and even after scrubbing (t (580.56) = -9.92, $p < 0.001$; Supplementary Fig. 4).

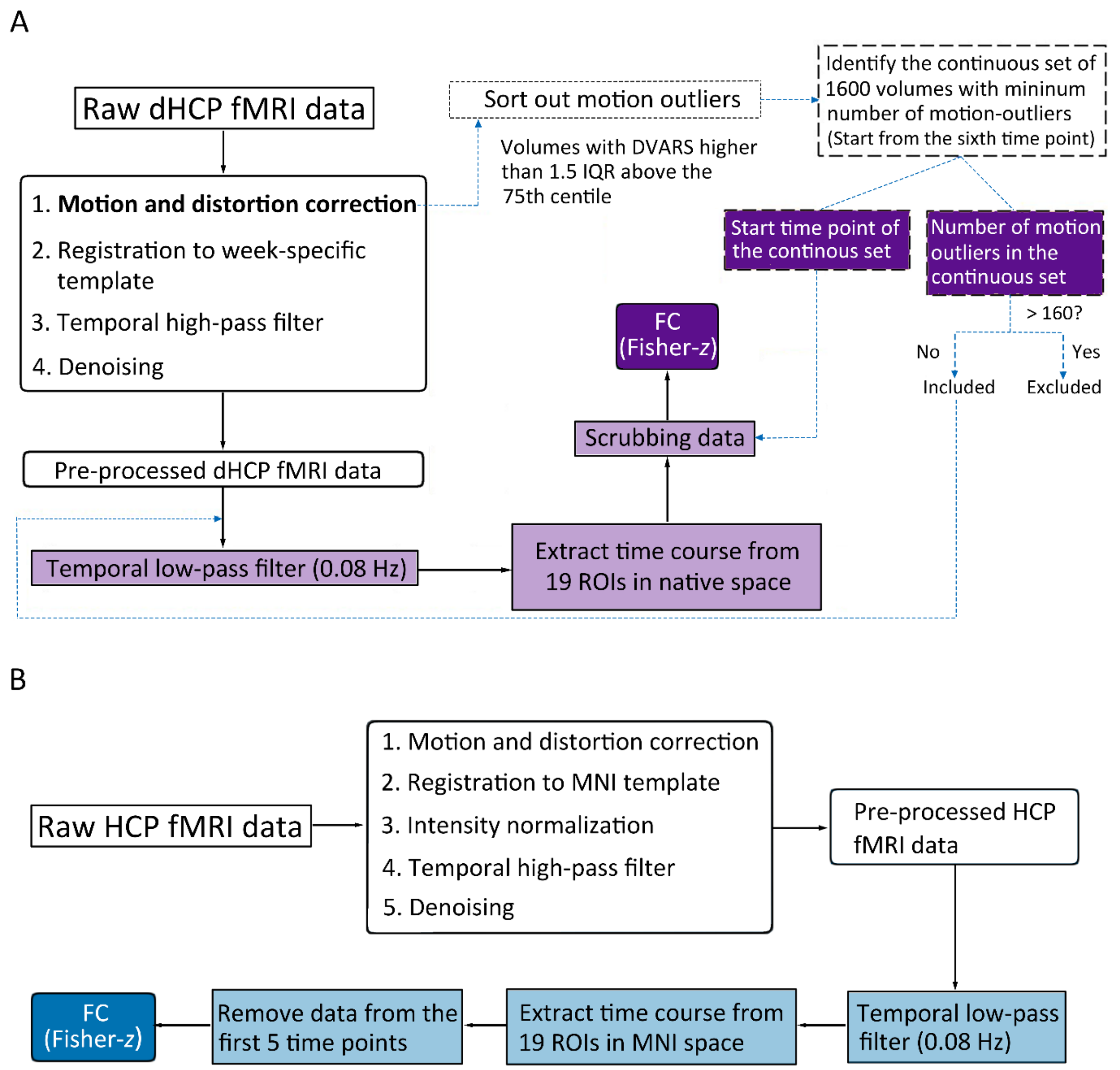

Supplementary Figure 1. Data processing steps for neonate and adult data. (A) Processing steps for the neonate rs-fMRI data. The rectangles with rounded corners indicate the steps in the fMRI neonatal pre-processing pipeline of the Developing Human Connectome Project. The frames in light purple indicate additional processing steps in this study, and the dotted black line rectangles represent the steps for correcting motion outliers. The frames in dark purple represent the data that we obtained. (B) Processing steps for the adult fMRI data. The rectangles with rounded corners indicate the steps in the fMRI pre-processing pipeline of the Human Connectome Project. The frames in light blue indicate additional processing steps in this study, and the frame in dark blue represents data that we obtained. Abbreviations: dHCP, developing Human Connectome Project; DVARS, D referring to temporal derivative of time courses and VARS referring to root mean squared variance over voxels; ROI, regions of interest; IQR, Inter Quartile Range; FC, functional connectivity; HCP, Human Connectome Project.

Network definition

We first aligned these ROIs with 40-week dHCP T1w template.[4] This involved: 1) trimming the dura from the 40-week dHCP T1w template, and the cerebellum from both the 40-week dHCP T1w template and MNI T1w template; 2) aligning the 40-week dHCP T1w template to MNI T1w template using non-linear registration (ANTs SyN) (See Supplementary Fig. 2 for the registration accuracy between them); 3) applying the warp file generated in the last step to the ROIs in MNI space with 40-week dHCP T1w template as a reference. In the next step, we needed to align these ROIs in 40-week dHCP T1w template space with neonate native space. We inverted the func-to-template warp provided by dHCP group and applied this inverted warp to ROIs in the 40-week dHCP T1w template space. Thus, we obtained ROIs in each neonate native functional space (Supplementary Fig. 3). For adults, as the denoised HCP data had been aligned to MNI space, we used these ROIs in MNI space directly.

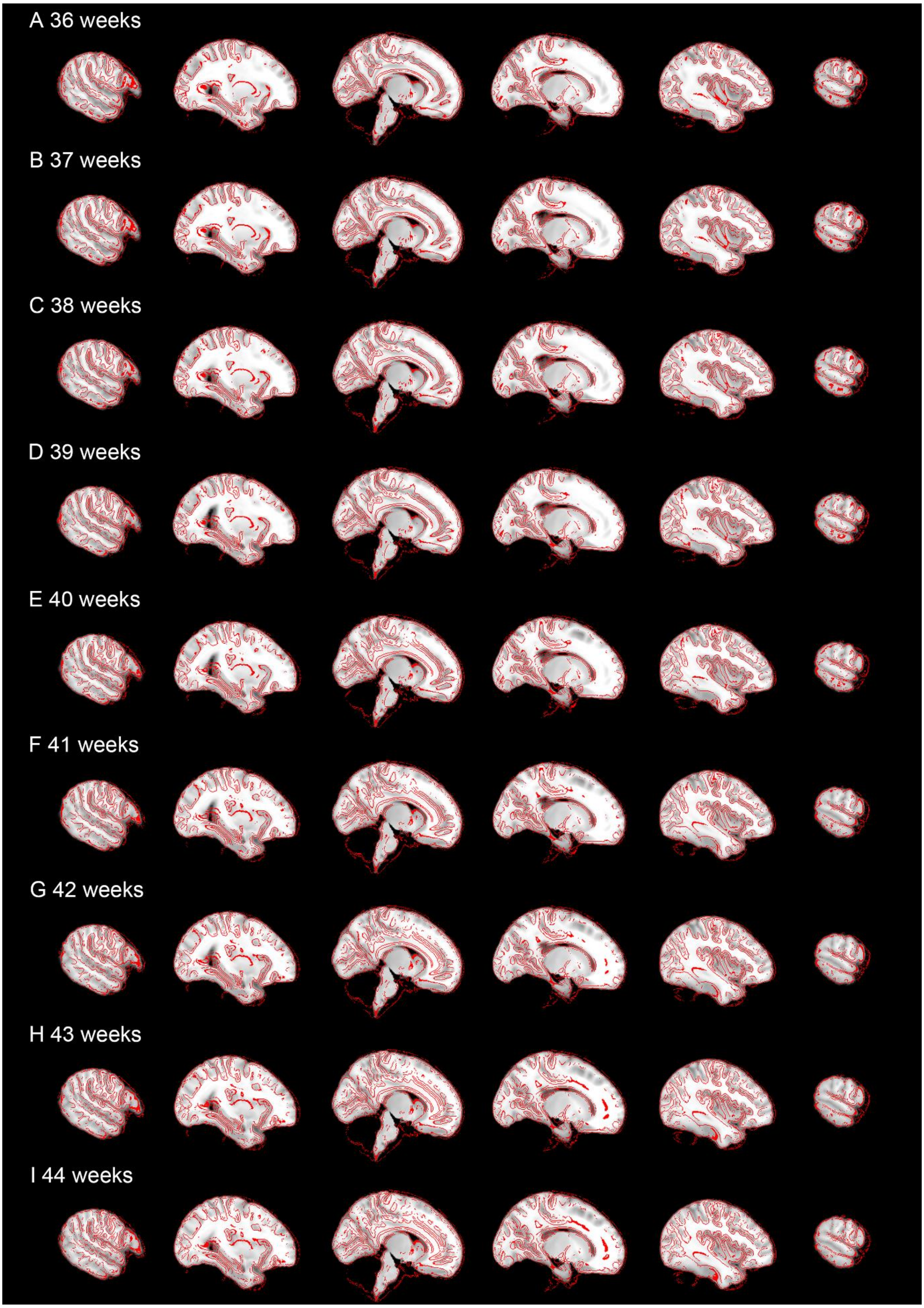

Supplementary Figure 2. Registration of the MNI T1 template to the 40-week dHCP T1w template. The 40-week dHCP T1w templates shown in red lines were overlaid on the MNI T1 template registered to the 40-week dHCP T1w template spaces.

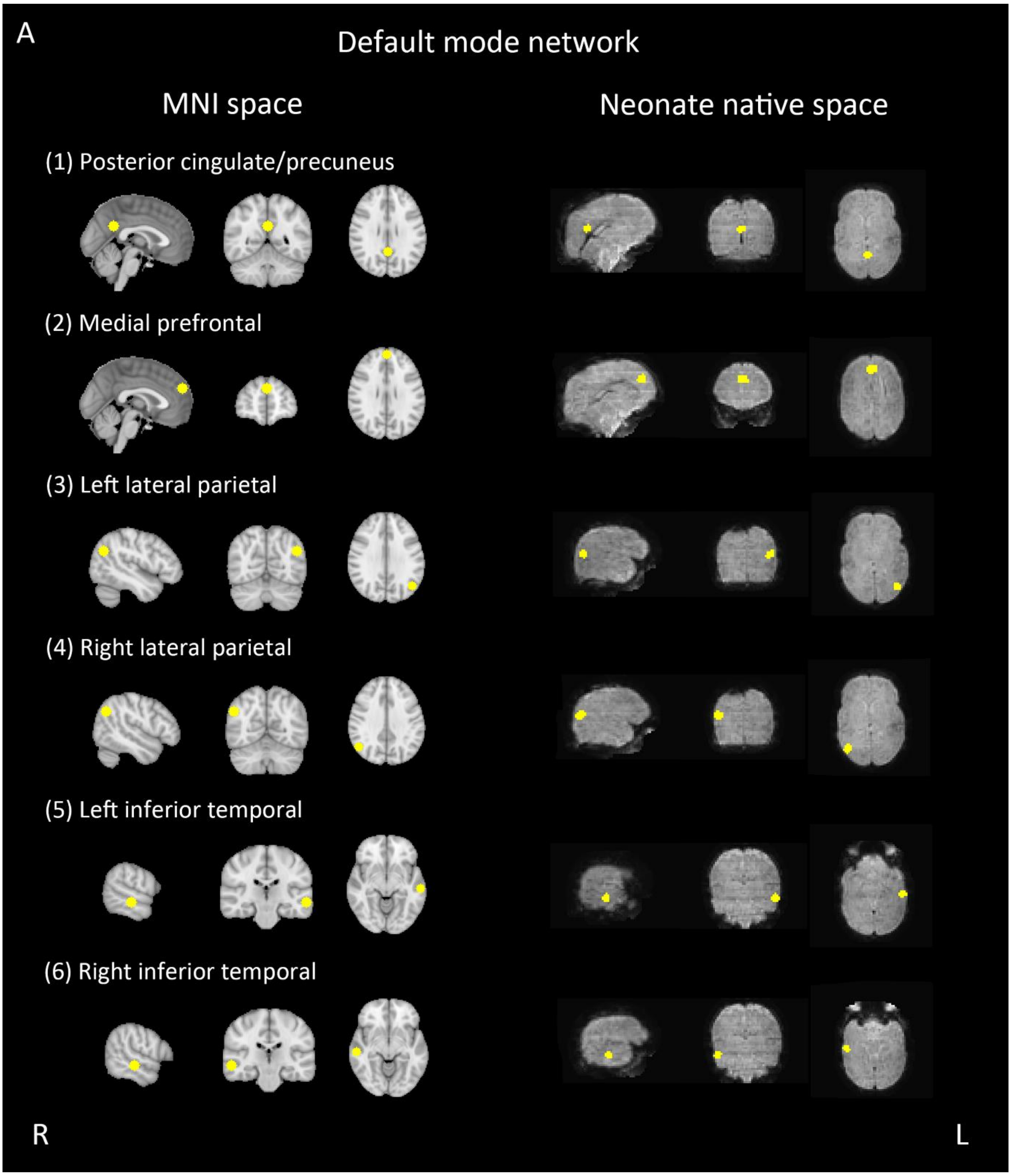
A
Default mode network
MNI space
Neonate native space
(1) Posterior cingulate/precuneus
(2) Medial prefrontal
(3) Left lateral parietal
(4) Right lateral parietal
(5) Left inferior temporal
(6) Right inferior temporal
R
L

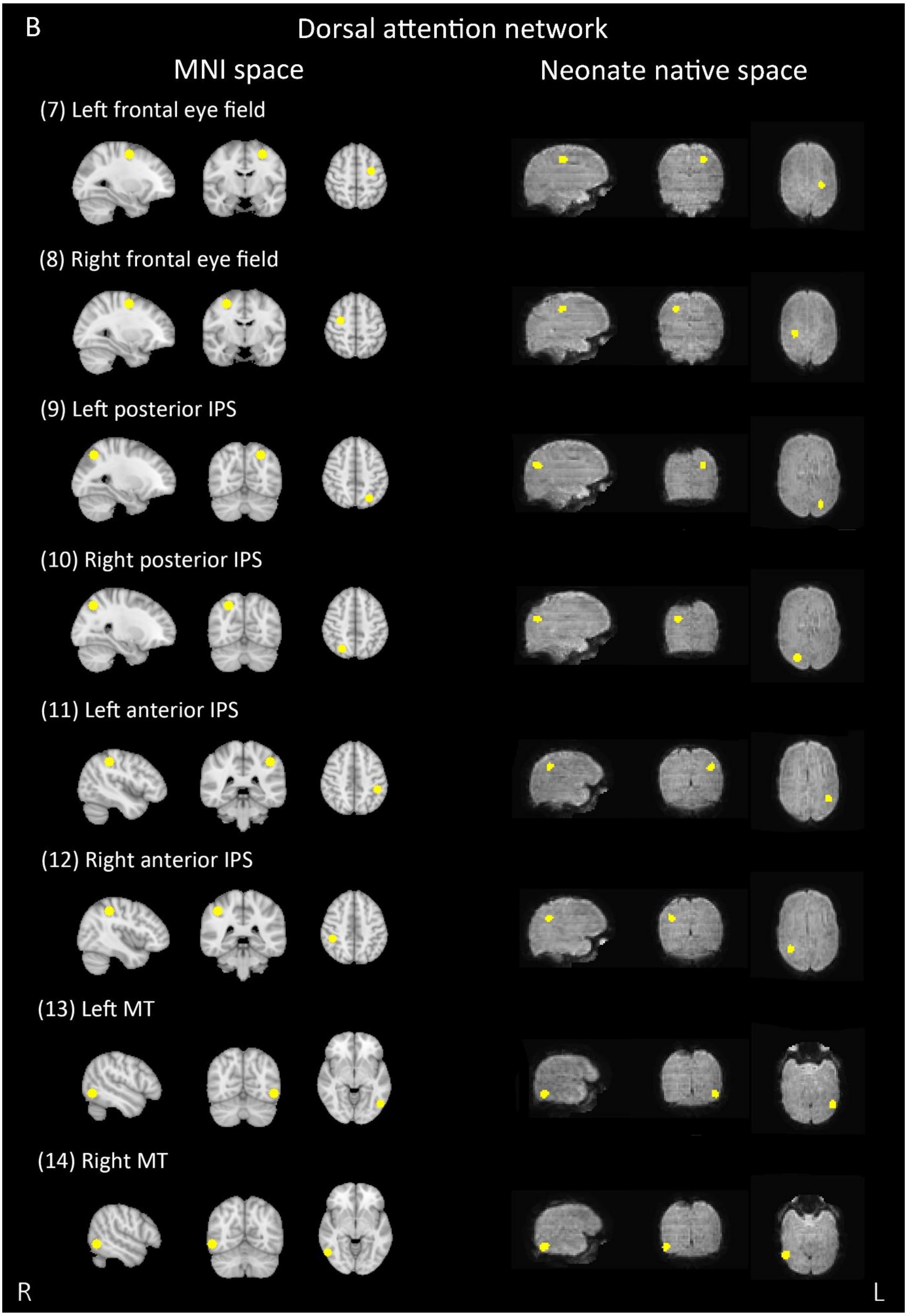
B
Dorsal attention network
MNI space
Neonate native space
(7) Left frontal eye field
(8) Right frontal eye field
(9) Left posterior IPS
(10) Right posterior IPS
(11) Left anterior IPS
(12) Right anterior IPS
(13) Left MT
(14) Right MT
R
L

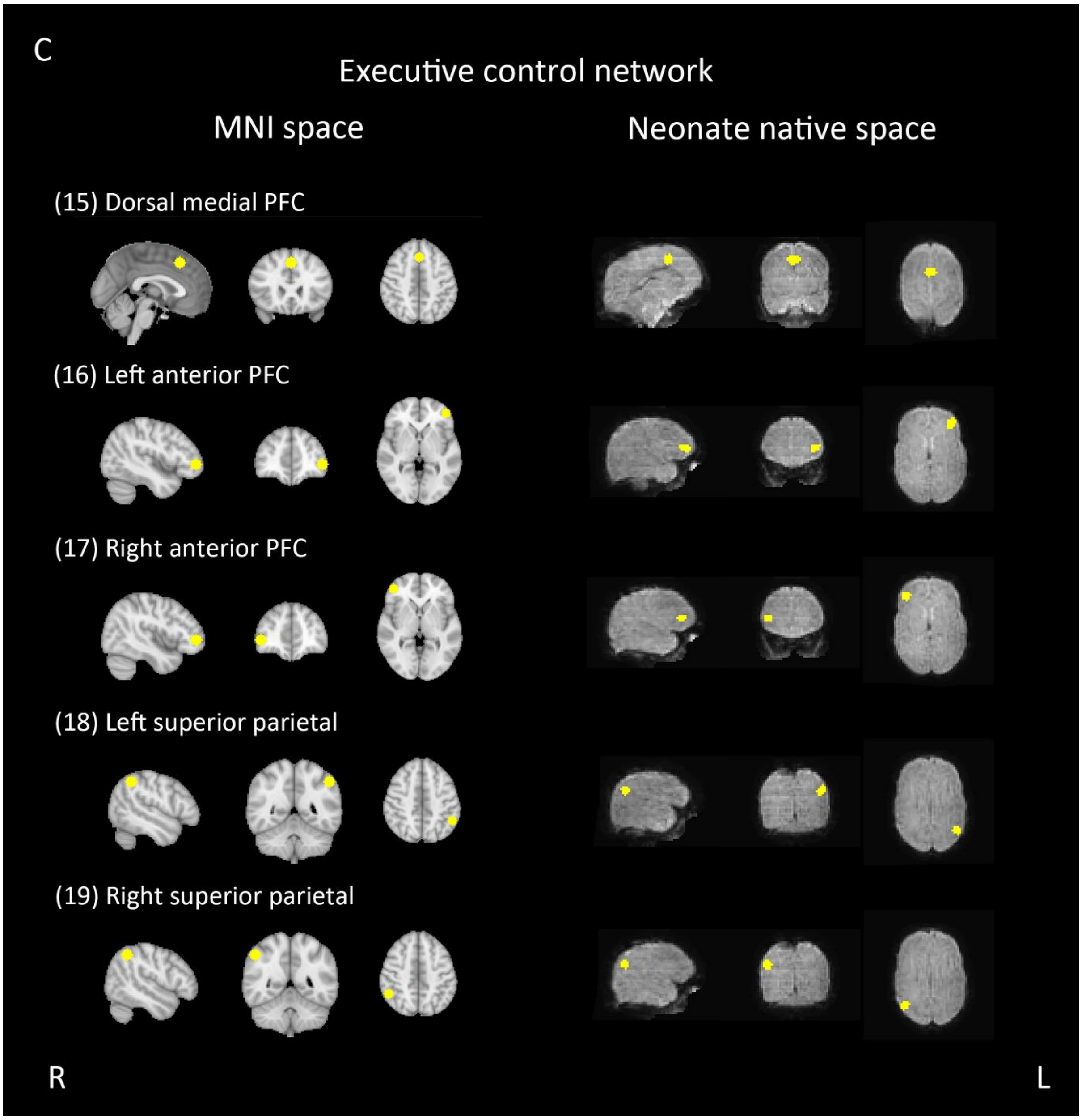

Supplementary Figure 3. The three networks in the MNI space and neonate native space. The functional images represented here were from one neonate (sub-CC00518XX15), which was randomly chosen. (A) default mode network, (B) dorsal attention network, and (C) executive control network. Abbreviations: IPS: Intraparietal sulcus, MT: Middle temporal area, PFC: Prefrontal cortex; R, right; L, left.

Data analyses

Hierarchical clustering analyses. We captured the structure of the three networks in different groups with hierarchical clustering analysis,[12,13] which has proven informative in prior studies.[14,15] This hierarchical clustering algorithm builds up an entire cluster tree in which neighbouring regions are joined if their similarity is maximal among all pairs of neighbouring regions. Here, we used the time-course extracted from the 19 ROIs as input to access the hierarchical relationship among the ROIs. For the neonate data, we first calculated initial pairwise distance between ROIs using one minus the linear correlation between the scrubbed time-courses extracted from the 19 ROIs at the individual level. For adults, the initial pairwise distance between ROIs was calculated using one minus the linear correlation between the time-courses of 1195 time points extracted from the 19 ROIs at the individual level. Then, we averaged the pairwise distances between ROIs within each group to get the group-level pairwise distances, which were submitted to hierarchical clustering analysis to create a hierarchical cluster tree of the 19 ROIs for each group respectively. The cophenetic correlation coefficient was used to create a dendrogram for each group. The length of each C link in the dendrogram represents the distance between regions/clusters.

Multidimensional scaling analysis. Non-metric multidimensional scaling (MDS) was also used to facilitate visualizing the similarity of ROIs functional response for adults and neonate groups. The non-metric MDS performs non-metric multidimensional scaling on the dissimilarity matrix of item−item (i.e., ROI−ROI dissimilarity matrix) to compute a configuration.[16] Then, the Euclidean distances between items (i.e., ROIs) in the configuration were obtained. The difference between the monotonic transformed dissimilarities in the item−item (i.e., ROI−ROI) matrix and the Euclidean distances between items (i.e., ROIs) in this configuration were minimized and items (ROIs) were represented in a low-dimensional

space (i.e., a 2-D space). The ROI−ROI dissimilarity matrix (one minus the linear correlation between the time-courses) for each group from the hierarchical clustering analysis was submitted to non-metric MDS analysis implemented in MATLAB.

Supplementary Results

Comparison of head motion in neonates and adults. Independent-sample *t*-tests were applied to compare the head motion in the adults and neonates before/after the scrubbing procedure. We found that adults had significantly lower head motion compared to neonates before (t (491.45) = -12.49, $p < 0.001$) and after (t (580.56) = -9.92, $p < 0.001$) scrubbing procedure (Supplementary Fig. 4). A paired-*t* test was applied to detect the difference in head motion in neonates before and after the scrubbing procedure. Results showed that neonates had significantly lower head motion after scrubbing relative to before scrubbing (t (427) = -9.69, $p < 0.001$) (Supplementary Fig. 4). Bonferroni correction for multiple comparisons was applied to statistical results.

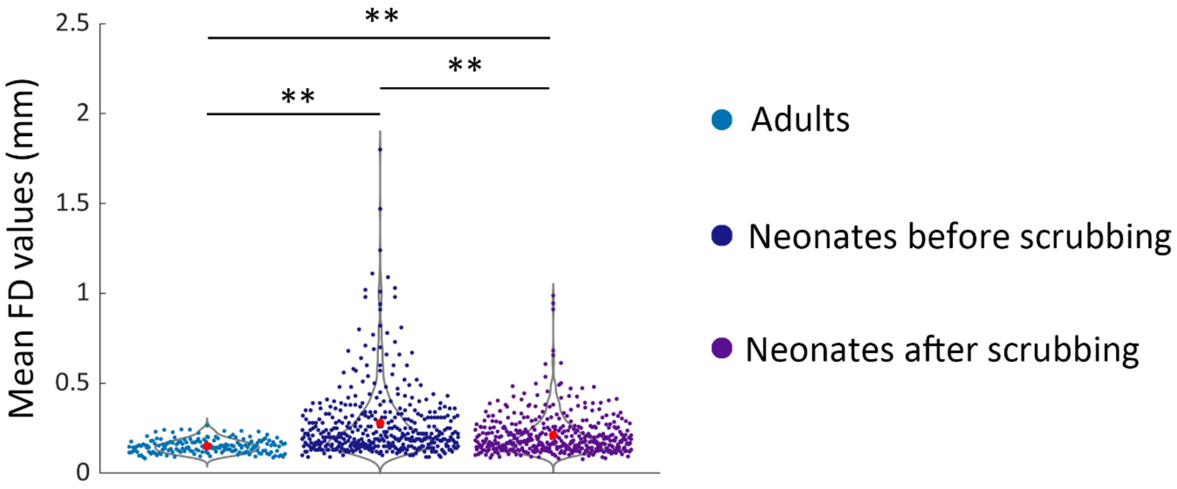

Supplementary Figure 4. Head motion in neonates and adults. The red dot indicates the mean framewise displacement value in each group. Independent-samples *t*-tests were applied to detect the difference between the adults and neonates. A paired-*t* test was applied to detect the difference between the neonates before and after scrubbing procedure. Abbreviations: FD, framewise displacement; ** = $p < 0.005$.

Comparison of head motion in neonate groups after scrubbing and adults. We conducted a one-way ANOVA to compare the difference in head motion between the adults and neonate groups after the scrubbing procedure and found a significant main effect of group (F (3, 600) = 16.46, $p < 0.001$). Independent-sample t-tests were applied to compare head motion between every two groups. We found that that adults had significantly lower head motion relative to the full-term neonates (t (368.44) = -8.67, $p < 0.001$), preterm neonates scanned at TEA (t (79.69) = -4.14, $p < 0.001$), and preterm neonates scanned before TEA (t (76.97) = -4.16, $p < 0.001$) (Supplementary Fig. 5). Bonferroni correction for multiple comparisons was applied to statistical results.

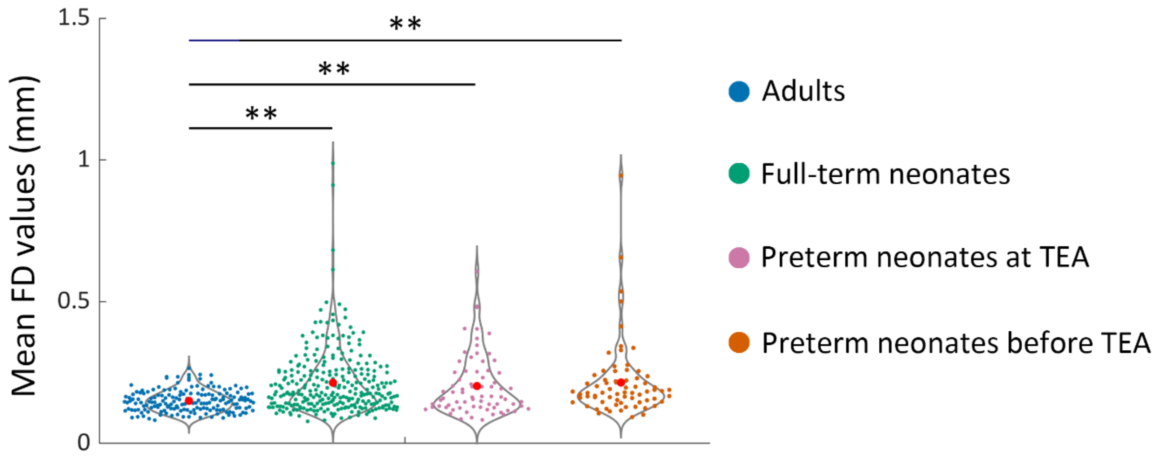

Supplementary Figure 5. Head motion in the neonate groups after censoring and the adults. The red dot indicates the mean framewise displacement value in each group. Independent-sample t-tests were applied to detect the difference between every two groups. Abbreviations: FD, framewise displacement; TEA, term-equivalent age; ** = $p < 0.005$.

High-order networks functional connectivity in adults. In adults, a 2 × 3 repeated measure ANOVA [*type of FC* (within-network, between-network) × *network* (DMN, DAN, ECN)] showed a significant main effect of type of FC (F (1, 175) = 2323.00, $p < 0.001$), which was driven by higher overall connectivity for the within- relative to between-network (t (175) =

48.11, $p < 0.001$). We also found a main effect of network (F (1.93, 337.773) = 37.50, $p < 0.001$), that was driven by lower overall connectivity for the DMN relative to the DAN (t(175) = -8.72, $p < 0.001$) and ECN (t (175) = -3.71, $p < 0.001$) and lower overall connectivity for the ECN relative to DAN (t (175) = -5.10, $p < 0.001$). Finally, a significant interaction effect of type of FC by network (F (2, 350) = 96.49, $p < 0.001$) was driven by a smaller within- vs between-network FC difference in ECN relative to DMN (t (175) = -11.20, $p < 0.001$) and DAN (t (175) = -13.03, $p < 0.001$). Paired-t tests showed significantly higher within- to between-network FC for each network (DMN: t (175) = 30.69, $p < 0.001$; DAN: t (175) = 42.00, $p < 0.001$; ECN: t (175) = 27.40, $p < 0.001$) (Supplementary Fig. 6), confirming that each of the three networks was differentiated as a cohesive unit in adults. Bonferroni correction for multiple comparison was applied to statistical results.

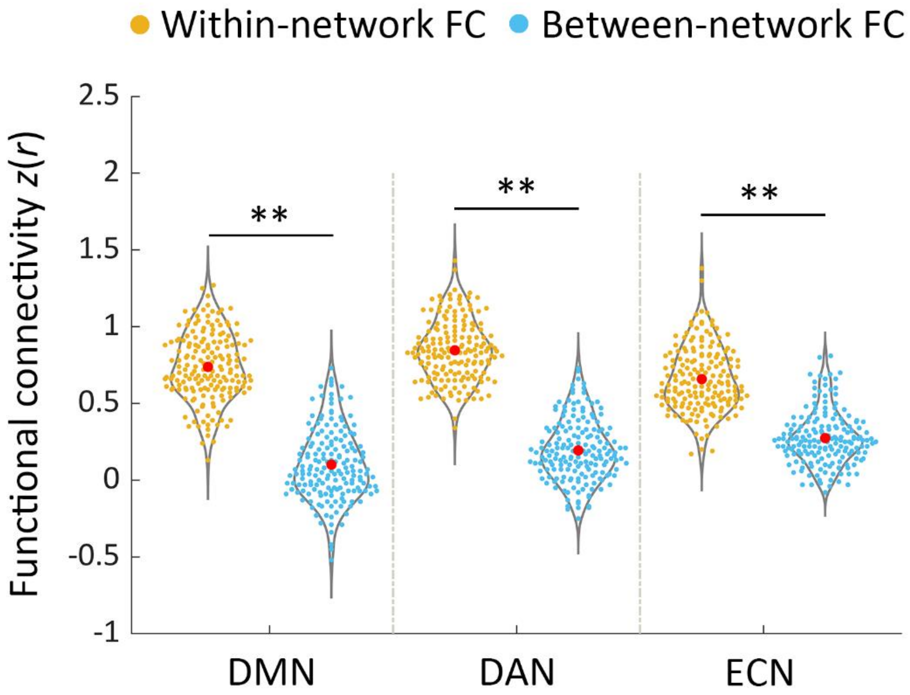

Supplementary Figure 6. Network functional connectivity in adults. The between-network connectivity depicts the average FC of each network with the other two. The red dot indicates the mean within/between-network functional connectivity of each network. Paired-t tests were applied to detect the difference between within-network and between-network FC for each network. Abbreviations: DMN, default mode network; DAN, dorsal attention network; ECN, executive control network; FC, functional connectivity; ** = $p < 0.005$.

The reciprocal relationship between the DMN and prefrontal networks in adults. A one-way ANOVA with repeated measures for between-network FC (DMN−DAN, DMN−ECN, DAN−ECN) showed a significant main effect (F (1.93, 337.22) = 118.92, $p < 0.001$), which was driven by significantly lower FC in the DMN−DAN relative to the DMN−ECN (t (175) = -6.19, $p < 0.001$) and DAN−ECN pairings (t (175) = -14.01, $p < 0.001$), and significantly lower FC in the DMN−ECN relative to DAN−ECN pairing (t (175) = -9.61, $p < 0.001$) (Supplementary Fig. 7). Bonferroni correction for multiple comparisons was applied to statistical results. The lower FC between the DMN and DAN relative to the other pairings suggested a reciprocal relationship between the two networks in adults.

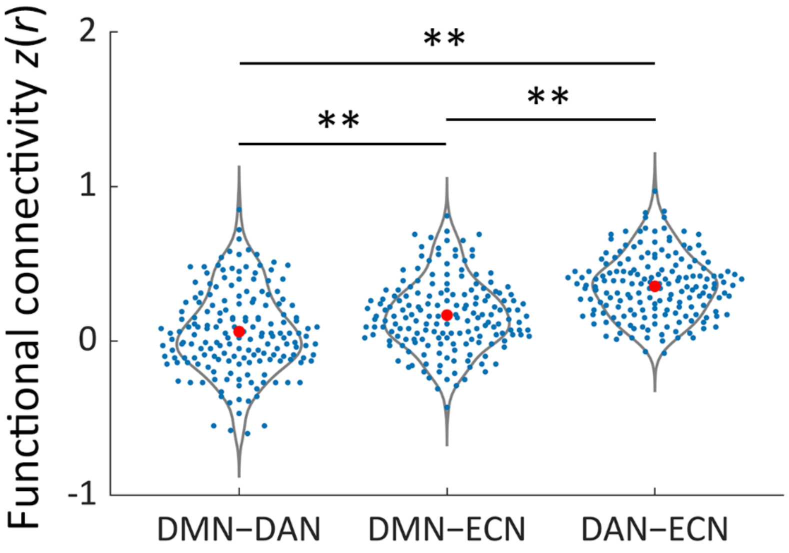

Supplementary Figure 7. Between-network functional connectivity (FC) in adults. FC values were Fisher-z transformed. The red dot indicates the mean functional connectivity in each network-pairing. Paired-*t* tests were applied to detect the difference between every two between-network FCs. Abbreviations: DMN−DAN, FC between the default mode network and dorsal attention network; DMN−ECN, FC between the default mode network and executive control network; DAN−ECN, FC between the dorsal attention network and executive control network; ** = $p < 0.005$.

Comparison of DMN-frontoparietal functional connectivity in neonates and adults. To investigate between-network connectivity in neonates relative to adults, we created a GLM that controlled for head motion, and compared each neonate group to the adult group. For full-term neonates, we found significant main effects of group for all of the pairings (DMN−DAN: F (1, 455) = 278.77, $p < 0.001$; DMN−ECN: F (1, 455) = 195.83, $p < 0.001$; DAN−ECN: F (1, 455) = 15.59, $p < 0.001$), which was driven by significantly lower FC in DMN−DAN and DMN−ECN, and higher FC in DAN−ECN in the adults related to full-term neonates (Fig. 7A). These results suggested that the DMN was more functionally differentiated from the DAN and ECN, and thus, suggesting a stronger reciprocal relationship in adults relative to full-term neonates. For preterm neonates scanned at TEA, we found a significant main effect of group for DMN−DAN (F (1, 246) = 105.83, $p < 0.001$), DMN−ECN (F (1, 246) = 91.39, $p < 0.001$), which was driven by significantly lower FC in DMN−DAN and DMN−ECN in the adults related to preterm neonates scanned at TEA (Fig. 7B). Similarly, to full-term neonates, these results demonstrated that the DMN was more functionally differentiated from DAN and ECN, suggesting a stronger reciprocal relationship in adults relative to preterm neonates scanned at TEA. We also compared between-network connectivity in preterm neonates scanned before TEA and adults using a GLM that controlled for head motion, although we did not observe a reciprocal relationship between DMN and frontoparietal network in that neonate group. We found a significant main effect of group for DMN−DAN (F (1, 246) = 257.78, $p < 0.001$) and DMN−ECN (F (1, 246) = 45.54, $p < 0.001$), which was driven by significantly lower FC in DMN−DAN and DMN−ECN in the adults relative to the preterm neonates scanned before TEA (Fig. 7C).

Supplementary Tables

Supplementary Table 1. Previous rs-MRI studies of brain network development in neonates

No.	Article	Subjects	GA at birth	Age at scan	State at scan	Scanner	TR (ms)	voxel size (mm^3)	Duration (min)	Head motion control	Template	Analysis method	Neural network identified
**1**	**Fransson et al.[17]**	12 preterm neonates scanned at TEA	25 weeks 6 days (24 weeks 4 days −27 weeks 5 days)	41 weeks 0 days PMA (39 weeks 1 day − 44 weeks 3 days)	under sedation	1.5 T	2000	2.8 × 2.8 × 4.5	10	scrubbing	neonate brain template[18]	ICA	1) VIS; 2) SMN; 3) AUD; 4) a network including the precuneus area, lateral parietal cortex, and the cerebellum; 5) an anterior network that incorporated the medial and dorsolateral prefrontal cortex.
**2**	**Fransson et al.[19]**	19 full-term neonates	38 weeks 4 days (37 weeks 4 days – 39 weeks 1 day)	40 weeks 2 days PMA (39 weeks 2 days – 41 weeks 6 days)	natural sleep	1.5 T	2000	2.8 × 2.8 × 4.5	10	scrubbing	neonate brain template[18]	ICA	1) VIS; 2) SMN; 3) bilateral temporal/inferior parietal cortex including the primary auditory cortex, 4)posterior lateral and midline aspects of the parietal cortex; 5) medial and lateral parts of the prefrontal cortex and 6) the bilateral basal ganglia.
3	Lin et al.[20]	38 preterm and full-term neonates	35 – 42 weeks	2 – 4 weeks after birth	natural sleep	3T	2000	4 × 4 × 4	5	scrubbing	data-specific template	SCA	SMN and VIS (exists as early as 2 weeks after birth)

		26 one-year-olds born prematurely or at full-term age	N/A	N/A									
		21 two-year-olds born prematurely or at full-term age	N/A	N/A									
**4**	**Doria et al.[21]**	17 early preterm neonates scanned before TEA	25 weeks 2 days – 31 weeks 1 day	29 weeks 0 days – 32 weeks 1 day PMA	natural sleep	3T	1500	2.5 × 2.5 × 3.25	6.5	FD > 5 mm	data-specific template	ICA and SCA	1) In full-term neonates and preterm neonates scanned at TEA: VIS, AUD, SMN, motor, DMN, FPN, and ECN were completely present;<br>2) In early preterm neonates: AUD, dorsal visual stream and ECN were not found;<br>3) Before TEA, the DMN was incomplete.
		21 preterm neonates scanned before TEA	26 – 35 weeks	33 weeks 0 days – 36 weeks 4 days PMA	natural sleep (17/23) /under sedation								
		24 preterm neonates scanned at TEA	24 weeks 3 days – 35 weeks 3 days	39 weeks 3 days – 43 weeks 2 days PMA	under sedation								
		8 full-term neonates	38 – 41 weeks 4 days	39 weeks 1 day – 43 weeks 4 days PMA	natural sleep (6/8) /under sedation								
**5**	**Smyser et al.[22]**	53 preterm neonates	23 weeks 2 days – 34 weeks 0 day	1) < 30 weeks PMA<br>2) 30 weeks PMA<br>3) 34.0 weeks PMA<br>4) 38.0 weeks PMA	natural sleep/resting quietly	3T	2910	2.4 × 2.4 × 2.4	10	scrubbing	Adult template	SCA	1) In preterm neonates and full-term neonates: motor-leg, motor-hand, motor-face, PCC, ACC, occipital, MPFC, LPFC, temporal, thalamus and cerebellum.<br>2) DMN precursor in full-term neonates.
		10 full-term newborns	N/A	Within 2—3 days of birth									

**6**	**Alcauter et al.[23]**	112 preterm and full-term neonates<br><br>129 one-year-olds<br><br>92 two-year-olds	35 – 42 weeks<br><br>N/A<br><br>N/A	4 weeks 5 days ± 2 weeks 5 days after birth<br><br>1 year 32 days ± 35 days<br><br>2 year 32 days ± 33 days	natural sleep	3T	2000	4 × 4 × 4	5	scrubbing	data-specific template	SCA	1) Neonates: Adultlike SMN, AUD, medial visual, and occipital pole and SN; Incomplete lateral visual, DMN, and FPN.<br>2) The lateral VIS, DMN, FPN showed dramatic synchronization during the first year and had minor refinement during the second year.
**7**	**Gao et al.[24]**	20 preterm and full-term neonates<br><br>24 one-year-olds<br><br>27 two-year-olds	35 – 42 weeks<br><br>N/A<br><br>N/A	3 weeks 3 days ± 1 week 5 days after birth<br><br>1 year 1 month ± 1 month<br><br>2 years 1 month ± 1 month	natural sleep	3T	2000	4 × 4 × 4	5	scrubbing	data-specific template	ICA	An incomplete DMN is present in 2-week-olds.<br>More adultlike DMN were found in 1-year-olds and 2-year-olds.
**8**	**Gao et al.[25]**	51 full-term and preterm neonates<br><br>50 one-year-olds<br><br>46 two-year-olds	35 – 42 weeks<br><br>N/A<br><br>N/A	3 weeks 2 days ± 1 week 5 days after birth<br><br>1 year 1 month ± 1 month<br><br>2 years ± 1 months	natural sleep	3T	2000	5 × 4 × 4	5	None	a subject not included in this study and scanned at 3 weeks	SCA	1) Neonates: incomplete DMN and DAN.<br>2) 1-year-olds: highly synchronized DMN and DAN.

**9**	**Gao et al.[26]**	65 full-term neonates	35 – 42 weeks	< 1 month (N = 45)<br>3 months (N = 34)<br>6 months (N = 33)<br>9 months (N = 29)<br>12 month (N = 35)	natural sleep	3T	2000	4 × 4 × 4	5	scrubbing	data-specific template and adult MNI template	SCA	1) Neonates: SMN, AUD, VN; incomplete lateral visual/parietal network, SN, DMN, FPN.<br>2) 1-year-olds: adultlike lateral visual/parietal network and DMN; incomplete SN and FPN
**10**	**Gao et al.[27]**	143 full-term neonates	36 – 42 weeks	4 weeks 5 days ± 2 weeks 5 days after birth (N = 112)<br><br>1 year old (N = 129)<br><br>2 years old (N = 92)	natural sleep	3T	2000	4 × 4 × 4	5	scrubbing	data-specific template	ICA	1) Neonates: VN and SMN;<br>2) The auditory/language, lateral visual/parietal, DMN, right FPN, SN, and left FPN were topologically incomplete and isolated in neonates but demonstrated consistent synchronization during the first 2 years of life.
**11**	**Wylie et al.[28]**	12 full-term neonates	N/A	8 weeks 0 days ± 2 weeks 6 days after birth	under sedation	3T	2000	3.43 × 3.43 × 3.4	N/A	motion larger than 1 voxel	MNI template	ICA	DMN, VIS, AUD, SMN, basal ganglia, precuneus, visual spatial, language, ECN, anterior SN.
12	Damaraju et al.[29]	4-month-old full-term infants<br><br>9-month-old full-term infants	N/A	18 weeks 4 days ± 2 weeks 1 day after birth<br><br>40 weeks 4 days ± 1 week 2 days after birth	natural sleep	3T	2000	thickness = 3.5 mm	8.3	despiking step	9-month MNI template[30]	ICA	In both groups: sub-cortical, AUD, VIS, SMN, DMN, temporal, attentional and frontal network, cerebellum.

**13**	**He et al.[31]**	27 preterm neonates scanned at TEA	26 weeks 6 days ± 2 weeks 0 days	39 weeks 4 days ± 1 week 3 days PMA	natural sleep	3 T	3000	slice thickness = 3 mm	5.2	scrubbing	neonate brain template[32]	ICA	FPN, ECN, motor, SMN, medial visual, occipital visual and lateral visual areas.
**14**	**He et al.[33]**	34 preterm neonates	≤ 32 weeks	32 weeks 4 days ± 1 week 0 days PMA (N = 19)<br><br>39 weeks 2 days ± 1 week 2 days PMA (N = 22)<br><br>52 weeks 6 days ± 1 week 4 days PMA (N = 25)	natural sleep	3 T	3000	slice thickness = 3 mm	5.2	scrubbing	neonate brain template[32]	ICA	In all the three groups: The occipital visual, medial visual, lateral visual, AUD, motor, SMN, cerebellum, brainstem, DMN, ECN and FPN.
15	Ball et al.[34]	105 preterm neonates scanned at TEA<br>26 full-term neonates	30 (23 – 34) weeks<br><br>39 (37 – 41) weeks	42 (39 – 48) weeks PMA<br><br>43 (39 – 46) weeks PMA	under sedation	3 T	1500	2.5 × 2.5 × 4	6.4	ICA + FIX clean up	preterm brain template[35]	ICA	For both groups: frontal, parietal, temporal, occipital cortex, basal ganglia and cerebellum.
16	Weinstein et al.[36]	32 preterm neonates	29 weeks 0 days ± 2 weeks 5 days	37 weeks 4 days ± 1 week 4 days PMA	natural sleep	3 T	3000	slice thickness = 3 mm	N/A	None	None	SCA	Motor and VIS
**17**	**Cui et al.[37]**	44 preterm neonates scanned before TEA	24 weeks 5 days – 32 weeks 2 days	32 weeks 1 day ± 1 week 5 days PMA (29 weeks 6 days – 35 weeks 4 days)	N/A	3 T	2000	4 × 4 × 4	N/A	None	Montreal Neurological Institute pediatric atlas	ICA	Medial visual, lateral visual, AUD, SN, motor, prefrontal network, brainstem and thalami, frontal cortical network and cerebellum.

**18**	**Linke et al.[38]**	11 preterm neonates (No neuropathology)	27 weeks 4 days (25 – 34)	37 weeks 0 days PMA (35 – 42)	natural sleep with playing lullabies	1.5 T	1920	3 mm isotropic resolution	7	average >2 mm in two or more of the four fMRI runs	UNC neonatal brain template[39]	ICA	For both preterm scanned at TEA and full-term neonates: motor, AUD, VIS, ECN and DMN.
		19 preterm neonates (Neuropathology)	27 weeks 4 days (24 – 36)	37 weeks 4 days PMA (35 – 41)									
		3 full-term neonates (No neuropathology)	40 weeks 0 days (39 – 41)	40.5 (40 – 41) weeks PMA									
		7 full-term neonates (Neuropathology)	40 weeks 0 days (38 – 41)	41.0 (39 – 43) weeks PMA									
**19**	**Rajasilta et al.[40]**	21 full-term neonates	39 weeks 6 days ± 1 week 1 day	3 weeks 5 days ± 1 week 0 days after birth	natural sleep	3T	2500	3 × 3 × 3	6	motion larger than 3 mm in multiple time points	UNC2 neonate T2 template[39]	ICA	VIS, AUD, thalamic, basal ganglia, cerebellar and brainstem, insular, SMN, motor, DMN, prefrontal, frontal, parietal and temporoparietal networks.
20	**Eyre et al., [41]**	24 full-term neonates	38 weeks 6 days – 42 weeks 0 days)	43 weeks 4 days – 44 weeks 3 days	Natural sleep	3 T	392	2.16 × 2.16 × 2.15	15	scrubbing	Week-specific template	ICA	Lateral motor, medial motor, SMN, VIS, AUD, prefrontal, FPN, motor association, posterior parietal, temporoparietal, and visual association.

Abbreviations: GA, gestational age; PMA, postmenstrual age; ICA, Independent Component Analysis; SCA, Seed Correlation Analysis; FD, framewise displacement; TEA, term-equivalent age; SMN, sensorimotor network; VIS, visual network; DMN, default mode network; AUD, auditory network; FPN, frontoparietal network; ECN, executive network; SN, salience Network; ACC, anterior cingulate cortex; PCC, posterior cingulate cortex; MPFC, medial prefrontal cortex; LPFC, lateral prefrontal cortex. The studies in bold are the most relevant to the current research, based on direct focus on the presence of the DMN/DAN/ECN and similar infant age range.

Supplementary Table 2 Previous findings on the presence of the three high-order networks in infancy.

Studies	DMN	DAN	ECN
**Fransson et al., [17]**	×	×	×
**Fransson et al., [19]**	×	×	×
Lin et al. [20]	-	-	-
**Doria et al., [21]**	√	√(FPN)	√
**Smyser et al., [22]**	√(precursor)	×	×
**Alcauter et al., [23]**	√(incomplete)	√ (incomplete FPN)	×
**Gao et al., [24]**	√(incomplete)	-	-
**Gao et al., [25]**	√(incomplete)	√(incomplete)	×
**Gao et al., [26]**	√(incomplete)	√ (incomplete FPN)	×
**Gao et al., [27]**	√(incomplete)	√ (incomplete FPN)	×
**Wylie et al., [28]**	√	×	√
Damaraju et al [29]	√	×	×
**He et al., [31]**	×	√(FPN)	√
**He et al., [33]**	√	√(FPN)	√
Ball et al., [34]	×	×	×
Weinstein et al., [36]	-	-	-
**Cui et al., [37]**	×	×	×
**Link et al., [38]**	√	×	√
**Rajasilta et al., [40]**	√	×	×
**Eyre et al., [41]**	×	√(FPN)	×

Abbreviations: DMN, default mode network; DAN, dorsal attention network; ECN, executive network; FPN, frontoparietal network. "√" indicates that network was identified while "×" indicates not; "-" means that network was not investigated in that study. The studies in bold are the most relevant to the current research, based on direct focus on the presence of the DMN/DAN/ECN and similar infant age range.

Supplementary Table 3. Information obtained from the developing Human Connectome Project for scans used in the present study.

Group	Sex	Birth age (GA, weeks ± days)	Scan age (PMA, weeks ± days)	Birth weight (Kg)
Full-term neonates	160M/122F	40.0 ± 8.6	41.2 ± 12.0	3.35 ± 0.54
Preterm neonates scanned at TEA	41M/32F	32.0 ± 25.6	40.9 ± 14.5	1.76 ± 0.79
Preterm neonates scanned before TEA	50M/23F	32.5 ± 13.4	34.6 ± 13.4	1.78 ± 0.61

Abbreviations: GA, gestational age; PMA, postmenstrual age; TEA, term-equivalent age; M, male; F, female.

Supplementary Table 4. Regions of interests for the default mode network, dorsal attention network and executive control network (from Raichle[42]).

Network	Index	ROI	MNI coordinates		
			x	*y*	*z*
**Default Mode Network**	1	Posterior cingulate/precuneus	0	-52	27
	2	Medial prefrontal	-1	54	27
	3	Left lateral parietal	-46	-66	30
	4	Right lateral parietal	49	-63	33
	5	Left inferior temporal	-61	-24	-9
	6	Right inferior temporal	58	-24	-9
**Dorsal Attention Network**	7	Left frontal eye field	-29	-9	54
	8	Right frontal eye field	29	-9	54
	9	Left posterior IPS	-26	-66	48
	10	Right posterior IPS	26	-66	48
	11	Left anterior IPS	-44	-39	45
	12	Right anterior IPS	41	-39	45
	13	Left MT	-50	-66	-6
	14	Right MT	53	-63	-6
**Executive Control Network**	15	Dorsal medial PFC	0	24	46
	16	Left anterior PFC	-44	45	0
	17	Right anterior PFC	44	45	0
	18	Left superior parietal	-50	-51	45
	19	Right superior parietal	50	-51	45

Abbreviations: IPS, Intraparietal sulcus; MT, Middle temporal area; PFC: Prefrontal cortex.

Supplementary references

1. Van Essen DC, Smith SM, Barch DM, *et al.* The WU-Minn Human Connectome Project: An overview. *Neuroimage*. 2013;80:62-79.

2. Ito T, Brincat SL, Siegel M, *et al.* Task-evoked activity quenches neural correlations and variability across cortical areas. *Plos Comput Biol*. 2020;16(8).

3. Fitzgibbon SP, Harrison SJ, Jenkinson M, *et al.* The developing Human Connectome Project (dHCP) automated resting-state functional processing framework for newborn infants. *Neuroimage*. 2020;223.

4. Schuh A, Makropoulos A, Robinson EC, *et al.* Unbiased construction of a temporally consistent morphological atlas of neonatal brain development. bioRxiv doi.org/10.1101/251512

5. Glasser MF, Sotiropoulos SN, Wilson JA, *et al.* The minimal preprocessing pipelines for the Human Connectome Project. *Neuroimage*. 2013;80:105-124.

6. Cusack R, Linke AC, Zubiaurre-Elorza L, *et al.* Differences in the spatial and temporal patterns of head motion during MRI of adults and infants. bioRxiv doi.org/10.1101/114447

7. Power JD, Mitra A, Laumann TO, Snyder AZ, Schlaggar BL, Petersen SE. Methods to detect, characterize, and remove motion artifact in resting state fMRI. *Neuroimage*. 2014;84:320-341.

8. Power JD, Barnes KA, Snyder AZ, Schlaggar BL, Petersen SE. Spurious but systematic correlations in functional connectivity MRI networks arise from subject motion. *Neuroimage*. 2012;59(3):2142-2154.

9. Sripada C, Rutherford S, Angstadt M, *et al.* Prediction of neurocognition in youth from resting state fMRI. *Mol Psychiatry*. 2020;25(12):3413-3421.

10. Gu S, Satterthwaite TD, Medaglia JD, *et al.* Emergence of system roles in normative neurodevelopment. *Proc Natl Acad Sci U S A*. 2015;112(44):13681-13686.

11. Hoptman MJ, Zuo XN, D'Angelo D, *et al.* Decreased interhemispheric coordination in schizophrenia: a resting state fMRI study. *Schizophr Res*. 2012;141(1):1-7.

12. Ripley BD, eds. *Pattern recognition and neural networks*. Cambridge university press; 2007.

13. Rasmussen E. Clustering algorithms. In: William B. Frakes RB-Y, eds. *Information retrieval: data structures and algorithms*. Prentice-Hall, Inc.; 1992:419–442.

14. Cusack R, Wild CJ, Zubiaurre-Elorza L, Linke AC. Why does language not emerge until the second year? *Hearing Research*. 2018;366:75-81.

15. Blumensath T, Jbabdi S, Glasser MF, *et al.* Spatially constrained hierarchical parcellation of the brain with resting-state fMRI. *Neuroimage*. 2013;76:313-324.

16. Kruskal JB. Nonmetric multidimensional scaling: a numerical method. *Psychometrika*. 1964;29(2):115-129.

17. Fransson P, Skiold B, Horsch S, *et al.* Resting-state networks in the infant brain. *P Natl Acad Sci USA*. 2007;104(39):15531-15536.

18. Dehaene-Lambertz G, Dehaene S, Hertz-Pannier L. Functional neuroimaging of speech perception in infants. *Science*. 2002;298(5600):2013-2015.

19. Fransson P, Skiold B, Engstrom M, *et al.* Spontaneous Brain Activity in the Newborn Brain During Natural Sleep-An fMRI Study in Infants Born at Full Term. *Pediatr Res*. 2009;66(3):301-305.

20. Lin W, Zhu Q, Gao W, *et al.* Functional connectivity MR imaging reveals cortical functional connectivity in the developing brain. *AJNR Am J Neuroradiol*. 2008;29(10):1883-1889.

21. Doria V, Beckmann CF, Arichi T, *et al.* Emergence of resting state networks in the preterm human brain. *Proc Natl Acad Sci U S A*. 2010;107(46):20015-20020.

22. Smyser CD, Inder TE, Shimony JS, *et al.* Longitudinal Analysis of Neural Network Development in Preterm Infants. *Cereb Cortex*. 2010;20(12):2852-2862.

23. Alcauter S, Lin W, Smith JK, *et al.* Development of thalamocortical connectivity during infancy and its cognitive correlations. *J Neurosci*. 2014;34(27):9067-9075.

24. Gao W, Zhu HT, Giovanello KS, *et al.* Evidence on the emergence of the brain's default network from 2-week-old to 2-year-old healthy pediatric subjects. *P Natl Acad Sci USA*. 2009;106(16):6790-6795.

25. Gao W, Gilmore JH, Shen DG, Smith JK, Zhu HT, Lin WL. The Synchronization within and Interaction between the Default and Dorsal Attention Networks in Early Infancy. *Cereb Cortex*. 2013;23(3):594-603.

26. Gao W, Alcauter S, Elton A, *et al.* Functional Network Development During the First Year: Relative Sequence and Socioeconomic Correlations. *Cereb Cortex*. 2015;25(9):2919-2928.

27. Gao W, Alcauter S, Smith JK, Gilmore JH, Lin WL. Development of human brain cortical network architecture during infancy. *Brain Struct Funct*. 2015;220(2):1173-1186.

28. Wylie KP, Rojas DC, Ross RG, *et al.* Reduced brain resting-state network specificity in infants compared with adults. *Neuropsychiatr Dis Treat*. 2014;10:1349-1359.

29. Damaraju E, Caprihan A, Lowe JR, Allen EA, Calhoun VD, Phillips JP. Functional connectivity in the developing brain: A longitudinal study from 4 to 9 months of age. *Neuroimage*. 2014;84:169-180.

30. Altaye M, Holland SK, Wilke M, Gaser C. Infant brain probability templates for MRI segmentation and normalization. *Neuroimage*. 2008;43(4):721-730.

31. He LL, Parikh NA. Aberrant Executive and Frontoparietal Functional Connectivity in Very Preterm Infants With Diffuse White Matter Abnormalities. *Pediatr Neurol*. 2015;53(4):330-337.

32. Kuklisova-Murgasova M, Aljabar P, Srinivasan L, *et al.* A dynamic 4D probabilistic atlas of the developing brain. *Neuroimage*. 2011;54(4):2750-2763.

33. He LL, Parikh NA. Brain functional network connectivity development in very preterm infants: The first six months. *Early Hum Dev*. 2016;98:29-35.

34. Ball G, Aljabar P, Arichi T, *et al.* Machine-learning to characterise neonatal functional connectivity in the preterm brain. *Neuroimage*. 2016;124(Pt A):267-275.

35. Serag A, Aljabar P, Ball G, *et al.* Construction of a consistent high-definition spatio-temporal atlas of the developing brain using adaptive kernel regression. *Neuroimage*. 2012;59(3):2255-2265.

36. Weinstein M, Ben-Sira L, Moran A, *et al.* The motor and visual networks in preterm infants: An fMRI and DTI study. *Brain Res*. 2016;1642:603-611.

37. Cui J, Tymofiyeva O, Desikan R, *et al.* Microstructure of the Default Mode Network in Preterm Infants. *Am J Neuroradiol*. 2017;38(2):343-348.

38. Linke AC, Wild C, Zubiaurre-Elorza L, *et al.* Disruption to functional networks in neonates with perinatal brain injury predicts motor skills at 8 months. *Neuroimage-Clin*. 2018;18:399-406.

39. Shi F, Yap PT, Wu G, *et al.* Infant brain atlases from neonates to 1- and 2-year-olds. *Plos One*. 2011;6(4):e18746.

40. Rajasilta O, Tuulari JJ, Bjornsdotter M, *et al.* Resting-state networks of the neonate brain identified using independent component analysis. *Developmental Neurobiology*. 2020;80(3-4):111-125.

41. Eyre, M., Fitzgibbon, S. P., Ciarrusta, J., Cordero-Grande, L., Price, A. N., Poppe, T., ... & Edwards, A. D. (2021). The Developing Human Connectome Project: typical and disrupted perinatal functional connectivity. *Brain*, 144(7), 2199-2213.

42. Raichle ME. The Restless Brain. *Brain Connectivity*. 2011;1(1):3-12.
